# Forest citizens and people‐centered conservation in the Brazilian Amazon

**DOI:** 10.1111/cobi.70031

**Published:** 2025-05-30

**Authors:** Luke Parry, Thiago F. Morello, James A. Fraser, Natalia Guerrero, Gabriela S. Lotta, Rodrigo C. Martins, Peter Newton, Jessica C. Pires Cardoso, Andreza A. Souza Santos, Mauricio Torres

**Affiliations:** ^1^ Lancaster Environment Centre Lancaster University Lancaster UK; ^2^ Amazonian Institute of Family Agriculture Federal University of Pará Belém Brazil; ^3^ Center of Modelling, Engineering and Applied Social Sciences Federal University of ABC São Paulo Brazil; ^4^ Land, Environment, Economics and Policy Institute University of Exeter Exeter UK; ^5^ Department of Public Administration Getulio Vargas Foundation São Paulo Brazil; ^6^ Department of Sociology Federal University of São Carlos São Carlos Brazil; ^7^ Department of Environmental Studies University of Colorado Boulder Colorado USA; ^8^ School of Global Affairs King's College London London UK

**Keywords:** democracy, demography, ecological citizenship, protected areas, rights, subcitizenship, traditional populations, áreas protegidas, ciudadanía ecológica, democracia, demografía, derechos, poblaciones tradicionales, sub‐ciudadanía, 权利, 次公民权, 民主, 生态公民权, 人口统计, 保护地, 传统居民

## Abstract

Demands for territorial recognition are foundational to the claiming of rights by forest‐proximate people who attempt to conserve their forests. The rights of these often‐marginalized populations have been largely overlooked by conservationists, yet they are central to achieving people‐centered conservation. We further developed the concept of forest citizenship as a normative framework and analytical tool based on Brazilian social environmentalism (*socioambientalismo*), *florestania* (a former political project in Acre state), Latin American scholarship on ecological citizenship, and Eurocentric political philosophy. Decades of struggle for territorial recognition and social inclusion have solidified the right to have rights for Amazonia's forest citizens. Hence, forest citizens are people who have become so through the sociopolitical dynamics of their rights claims. Forest citizenship is built on community mobilization to create legally recognized territories with participatory governance but becomes tangible only if individuals and communities can successfully claim other rights from institutions through everyday practices of citizenship. We also assessed the current number and distribution of forest citizens across Brazilian Amazonia based on gridded population data and spatial analyses to calculate the resident population in four territorial categories that meet these democratic preconditions: Indigenous lands, extractive reserves, sustainable development reserves, ecological settlement projects, and Afro‐descendent *Quilombola* territories. The territories covered 31% of the Legal Amazon, were home to 1.05 million forest citizens, and had diverse primary policy objectives but shared goals of empowering communities and conserving forests. To be emancipatory, forest citizenship must be bottom‐up, socially inclusive, and improve people's lives. We suggest that conservationists pay greater attention to power relations and decision‐making structures related to forest territories. Territory‐based forest citizenship may be relevant for other countries where environmentalism has intersected with struggles for land rights and democracy.

## Abstract (es)

Ciudadanía del bosque y conservación centrada en las personas de la Amazonia brasileña

**Resumen:**

Las demandas de reconocimiento territorial son fundamentales para la reivindicación de derechos por parte de las poblaciones cercanas a los bosques que intentan conservarlos. Los derechos de estas poblaciones, a menudo marginadas, han sido ignorados en gran medida por los conservacionistas, pero son fundamentales para lograr una conservación centrada en las personas. Desarrollamos el concepto de ciudadanía forestal como marco normativo y herramienta analítica con base en el socio ambientalismo brasileño, la *florestanía* (un proyecto político previo del estado de Acre), los estudios latinoamericanos sobre ciudadanía ecológica y la filosofía política eurocéntrica. Décadas de lucha por el reconocimiento territorial y la inclusión social han consolidado el derecho a tener derechos de los ciudadanos de la selva amazónica. Por lo tanto, los ciudadanos de los bosques son personas que se han convertido en tales a través de la dinámica sociopolítica de sus reivindicaciones de derechos. La ciudadanía forestal se construye sobre la movilización comunitaria para crear territorios legalmente reconocidos con una gobernanza participativa, pero sólo se hace tangible si los individuos y las comunidades pueden reclamar con éxito otros derechos a las instituciones a través de prácticas cotidianas de ciudadanía. También evaluamos el número actual y la distribución de los ciudadanos de los bosques en toda la Amazonia brasileña con base en datos de población cuadriculados y análisis espaciales para calcular la población residente en cuatro categorías territoriales que cumplen estas condiciones democráticas previas: tierras indígenas, reservas extractivas, reservas de desarrollo sostenible, proyectos de asentamientos ecológicos y territorios *quilombolas* afrodescendientes. Los territorios abarcaron el 31% de la Amazonia Legal, albergaron a 1.05 millones de ciudadanos de los bosques y tenían objetivos políticos primarios diversos, pero compartían las metas de empoderar a las comunidades y conservar los bosques. Para ser emancipadora, la ciudadanía forestal debe ser ascendente, socialmente inclusiva y mejorar la vida de las personas. Sugerimos que los conservacionistas presten más atención a las relaciones de poder y a las estructuras de toma de decisiones relacionadas con los territorios forestales. La ciudadanía forestal basada en el territorio puede ser relevante para otros países en los que el ambientalismo se ha cruzado con las luchas por los derechos sobre la tierra y la democracia.

## 巴西亚马逊地区的森林公民与以人为中心的保护

**【摘要】** 对于居住在森林附近并试图保护自己的森林的人群来说, 要求得到领地承认是其主张权利的基础。保护工作者很大程度上忽视了这些往往被边缘化的人群的权利, 但他们是实现以人为中心的保护的核心。我们在巴西社会环境主义(*socioambientalismo*)、*florestania*(阿克里州之前的一个政治计划)、拉丁美洲生态公民权学术研究, 以及欧洲中心主义政治哲学的基础上, 进一步发展了森林公民权的概念, 以将其作为一个规范框架和分析工具。数十年来, 争取领地承认和社会包容的斗争巩固了亚马逊森林公民所获得的权利。因此, 森林公民的身份是通过其权利诉求的社会政治动态而确立的。森林公民权建立在社区动员的基础上, 通过参与式治理来建立法律上承认的领地, 但只有当个人和社区能够通过日常行使公民权而成功从管理机构主张其他权利时, 森林公民权才有实际意义。

我们还利用网格人口数据和空间分析, 估计了巴西亚马逊地区森林公民的当前数量和分布情况, 并计算出符合这些民主先决条件的四类领地的常住人口数量, 领地包括:土著土地、采掘保护区和可持续发展保护区、生态定居计划, 以及非洲裔逃亡奴族群(*Quilombola*)领地。这些领地占法定亚马逊地区的31%, 居住着105万森林公民, 它们有不同的主要政策目标, 但都以加强社区能力和保护森林为共同目标。森林公民权必须是自下而上的, 具有社会包容性, 并能改善人们的生活, 这样才具有解放性。我们建议保护工作者应更加关注与森林领地相关的权力关系和决策结构。基于领地的森林公民权可能还适用于其他环保主义与争取土地权利和民主的斗争相交织的国家。**【翻译:胡怡思;审校:聂永刚】**

## INTRODUCTION

We sought to revitalize the terms *forest citizenship* (a sociopolitical process) and *forest citizen* (status of possessing forest citizenship) as a way of seeing forest peoples as rights holders. Schmink (2011), Schmink, et al. (2014), Mathews & Schmink (2015), and others have demonstrated that citizenship is a useful overarching concept for recognizing the rights of forest dwellers in the Global South. A citizenship perspective can provoke questions, such as, is a particular community conservation initiative sufficiently democratic (i.e., involving accountability and genuine participation)? How can complex socioenvironmental problems be addressed in socially inclusive ways? Are diverse peoples able to generate, refute, and modify conservation policies and projects? Is there space for protest, negotiation, and conflict resolution? These questions are profoundly important in Brazil, which reembraced democracy in the 1980s, developed a distinct social‐environmentalism from the 1990s (de Castro, 2012), saw a flourishing of democratic politics under President Lula da Silva (Lula) (2003–2011), and attacks on democracy under President Bolsonaro (2019–2022). Brazil remains deeply socially unequal, and people have little trust in public institutions (Pereira, 2020). More broadly, the challenge for environmental governance in Latin America is “the lack of a democratic tradition that favors relations between state and society based on the recognition of the latter as subject of rights” (Mora, 2023:862). Mora (2023) argues that Latin American countries are characterized by a situation of differentiated citizenship, where rights tend to be realized for the wealthier classes in certain core regions. For the rest of the population, especially those from poor and peripheral regions, including much of the Amazon rainforest, rights are often not realized (i.e., people are unable to exercise their rights). Hence, they have to struggle to access rights, in particular to land and public policies, and use forms of “insurgent citizenship” (i.e., rights claims from below) (Holston, 2008).

We sought to contribute to people‐centered conservation in Brazil, with a focus on the rights of Amazonian forest dwellers, in two ways. First, we further developed the concept of forest citizenship as a normative framework and analytical tool, defined as the claiming of rights by people who have achieved legal territorial recognition and who attempt to conserve their forests. Second, we quantified the number of forest citizens residing in different territorial categories in the Brazilian Amazon. This involved identifying and examining which territorial categories met the foundational criteria for forest citizenship. We then estimated the resident population of forest citizens in these territories. Finally, we drew on existing literature and unpublished documents to examine the diverse territories’ creation processes across several decades and to identify major environmental threats and ongoing rights struggles.

## METHODS

### Conceptualizing forest citizenship

Through the process of claiming territorial rights some of Amazonia's forest‐proximate people have already become forest citizens. Our conceptualization of forest citizenship builds on Brazilian social environmentalism (*socioambientalismo*), *florestania* (a former political project in Acre state), Latin American scholarship on ecological citizenship, and Eurocentric political philosophy. Wittman (2010:282) contends ecological citizenship emerges from Latin American concerns with social exclusion and inequality as “the discursive and active practice of relating the daily concerns of individual or family survival to that of the surrounding community and environmental space.” We believe that forest citizenship in Amazonia already exists, having emerged through the historical struggles of social movements, and has helped guarantee some forest dwellers’ rights and democratize conservation in Brazil.

In democracies citizenship is supposed to guarantee people's social, political, and civil rights. Citizenship refers to someone's status as a rights‐bearing member of a political community and this person's set of relationships with state institutions (Staeheli 2010). However, people's rights are not always recognized (i.e., acknowledged and upheld) in state‐citizenship relationships. In Brazil and elsewhere, traditional forest dwellers often experience rights deprivation and differentiated citizenship (Mathews & Schmink, 2015), which Holston (2008:4) defines as a “resilient regime of legalized privileges and legitimated inequalities” based on differences in property, race, gender, and occupation. The social and historical backdrop is Amazonia's reshaping by capital as a space of expropriation (plunder of work, nature, and care) as opposed to exploitation (extraction of surplus value from wage labor) (Fraser, 2022). Ongoing deforestation and smallholder land expropriation is interpreted as a deliberate form of violence, legitimized by former President Bolsonaro and others to improve productivity via private land appropriation and capital investment. The Brazilian state's history of selective noncompliance with law enforcement and justice in Amazonia (Barca & Milanez, 2021) demonstrates why ecological citizenship is relevant to achieving long‐term people‐centered conservation. In Latin America, ecological citizenship is about promoting instruments to ensure participation, information access, and social control over natural resources in relation to environmental rights and obligations (Gudynas, 2009). Environmental pressure from Amazonian communities has helped shape Brazilian statecraft and environmental politics, referred to by Hecht (2011) as “nation‐building from below.”

Sustainable use reserves (SURs) exemplify socioambientalismo, that is, societal attempts to jointly meet social and environmental goals under Brazil's 1988 democratic constitution (de Castro, 2012). Such legally protected, inhabited territories help conserve forests and biodiversity in the Brazilian Amazon (Gonçalves‐Souza et al., 2021; Herrera et al., 2019) and represent federal and state governmental responses to the protracted, politicized rights struggles of forest dwellers (Fraser, 2018). Socioambientalismo emerged because forest dwellers incorporated environmentalist discourse into their social justice agendas, including rubber‐tappers resisting violent dispossession by cattle ranchers (Hochstetler & Keck, 2007) and river dwellers resisting external threats, such as commercial fishing by outsiders (Aleixo & ATAMP 2011). Indigenous and traditional peoples asserted their forest cultures and identities to gain territorial rights under constitutional Article 1968, which transformed (in legal terms) squatters into citizens (Hecht, 2011). In his first two terms (2003–2010), President Lula emphasized social inclusion (Pereira, 2020) and territorial expansion, including 29 new extractive reserves (RESEXs) covering over 69,000 km^2^ (Gomes et al., 2018). The variety of territorial categories reflects Brazilian cultural diversity. These territories grant distinct social groups different territorial rights under the jurisdiction of different government agencies (Vega et al., 2022). De Castro (2012) reasons that territory‐based environmental governance in Brazil led to the emergence of environmental (or ecological) citizenship in Indigenous lands (ILs), SURs, agrarian reform settlements, and Quilombola territories (QTs).

Our notion of forest citizenship builds on florestania, a former political project in Acre state. This neologism was coined from *forest* and *citizenship* by Acre's so‐called Forest Government (a series of governors and their administrations from 1999 to 2018) to describe an ethical vision of development based on sustainable forest livelihoods. *Florestania* emerged from rubber tapper movements in the 1970s and 1980s and focused on socially inclusive, market‐oriented strategies for forest resource extraction, participation, and citizenship (Schmink et al., 2014). This differs from our territorial emphasis, albeit our conceptualization echoes *florestania's* “intention of extending citizenship to previously excluded forest residents” (Schmink, 2011). Florestania was explicitly political, fostering a “political belonging to the forest” and a “place‐based collectivity” (Latta & Wittman, 2010), drawing on Acriano history and cultural identity (Schmink, 2011). Yet florestania was dependent on a political cadre whose power waned and whose messaging refocused on “sustainable agro” (Pontes, 2022). Moreover, despite Schmimk's (2011) insistence that florestania addressed the goals, rights, and obligations for forest‐based development in Acre, the concept was never fully theorized. Finally, whereas florestania envisions forest dwellers as partners (Schmink, 2011), we consider forest dwellers as potential sources of resistance, opposition, and critique of misgovernance.

Forest citizenship can be seen as a form of Latin American ecological citizenship (Latta & Wittman, 2012), where major environmental challenges are “defined by the ecological dimensions of social, cultural and economic marginalization and injustice…the politics of nature are closely interwoven with struggles for recognition and inclusion…” (Latta & Wittman, 2010). Grassroots movements in Peru have exercised ecological citizenship to demand greater inclusion, access to information, and accountability for elites in decision‐making about natural resource use and misuse (Pieck, 2013). In the Global North, ecological citizenship's central focus has been greening democracy through people accepting their environmental duties and responsibilities (Dobson, 2003) and acting on them in their daily lives through practices of citizenship (Wolf et al., 2009). Moreover, the northern notion of ecological citizenship focuses on individual agency (ecological citizens becoming synonymous with sustainable consumers [MacGregor, 2014]) and largely ignores ways in which someone's ability to act is constrained by social, economic, cultural, and institutional contextual factors (Saíz, 2005). Nonetheless, for Amazonians living in legally recognized forest territories, rights are interconnected with environmental responsibilities (de Castro, 2012).

Forest citizenship is, then, the claiming of rights by people who have achieved legal territorial recognition and who attempt to conserve their forests. This particular form of ecological citizenship is based on the right to have rights: forest dwellers have to, first, collectively struggle for territorial recognition in order to, second, claim diverse rights. Inspired by Lund & Eilenberg (2017), we applied Somers’ (2008) two‐step conceptualization of citizenship—drawing on Arendt (1951): first, specify demands that individuals and groups of Amazonian forest dwellers be recognized by different state and multilateral institutions and included in Brazil's political community in order that they can claim rights and, second, claim bundles of rights enshrined in the 1988 constitution through everyday practices of citizenship.

Territorial recognition is necessary to achieve forest citizenship, given the link between control over land and the exercise of citizenship rights in Brazil (Wittman, 2010). Nonetheless, forest citizenship becomes tangible only if people who strive to become forest citizens can successfully claim rights from state and nonstate institutions through everyday practices of citizenship. De Castro (2012) emphasizes community involvement in territorial design and implementation, including diverse institutional collaborations and conflicts. Therefore, we propose that forest citizenship is restricted to territories that are created through community mobilization and later have participatory governance. Beyond claiming their basic rights and making their voices heard in conservation projects, the inhabitants of these territories must collectively fight for territorial survival by resisting land grabbing, periods of political indifference, and violence (Barca & Milanez 2021). Speaking to Chigudu's (2019) analysis of citizenship and cholera in Zimbabwe, forest citizenship is meaningful only if it is substantive and goes beyond political rights and social recognition to include improved access to high‐quality public services. We contend that forest citizens claim rights through grassroots movements and through participatory governance within existing state institutions (Pickering et al., 2020).

### Identifying and examining territorial categories foundational to forest citizenship

We identified legally recognized state and federal territorial categories in the Brazilian Amazon that fulfilled two criteria: were created (or being created) through bottom‐up mobilization and written manifestation of community territorial demands and, later had some level of participatory governance (i.e., during implementation). This analysis was based on scrutinizing management plans and other policy documents in Amazonas State (the site of most of our prior and ongoing fieldwork); our extensive Amazonian fieldwork experience; and our team discussions on forest citizenship in theory and practice since April 2022 (Section S9.1 in the Supporting Information Appendix). These sources also informed our conceptualization of forest citizenship. We excluded all categories of strictly protected areas and types of sustainable‐use territories where we considered that people were merely tolerated (rather than acknowledging dignity, worth, and identities) by state institutions (Brown, 2006).

We focused on four categories of forest citizen territories: ILs; SURs, including RESEXs and sustainable development reserves (RDSs); environmentally differentiated agrarian reform settlements, including agroextractivist settlement projects (PAEs), forestry settlement projects, and sustainable development projects (hereafter collectively ecological settlement projects [ESPs]); and QTs. We analyzed the creation and implementation processes of 46 territories and the justification for their creation (including higher‐level strategic objectives of each category and specific threats facing particular rural populations). We examined ongoing challenges that populations in particular territories face, related to category‐specific institutional processes and the rights‐claim challenges common to rural Amazonians. We did this by analyzing the management plans and assessments of the Instituto Socioambiental, a prominent Brazilian socioenvironmental nongovernmental organization (NGO). Our purpose was to identify ways in which practices of citizenship are enacted in the daily lives of forest citizens.

The selected territorial categories were partly justified by *regularização fundiária* (land regularization), which involves resolving insecure land tenure whose impacts include barriers to accessing credit for farming and housing (PAEs). Other higher level aims included ensuring the right to land (RESEXs); ensuring sustainable forest livelihoods and autonomy (RESEXs, PAEs); improving forest dwellers’ quality of life (RESEXs in Amazonas, PAEs); conserving high‐biodiversity areas (RDSs in Amazonas); and forest management (i.e., selective logging) (some RDSs in Amazonas). These aims were in response to diverse threats perceived by rural communities, government agencies, or nongovernmental partners (see “Territorial Creation Processes, Threats, and Ongoing Rights Struggles”). We excluded state and federal Forests (*Florestas*) because traditional river dwellers are merely tolerated (Section S1.3 in the Supporting Information Appendix). Despite cultural and livelihood commonalities between communities in Floresta and the SUR categories, Florestas are principally created to safeguard strategically important natural resource stocks, such as minerals and timber, or to attempt to limit the spatial influence of large‐scale commercial operations in Amazonia. Floresta management includes commercial‐scale extraction, such as logging, with community involvement.

### Assessing population size and distribution of forest citizens

We assembled shapefiles of the relevant territorial categories. We included only territories that (in or before 2022) had either completed the legal creation process or were sufficiently advanced to mean communities had probable de jure territorial rights in the case of legal disputes (Appendix S3). We used ArcGIS (ESRI) software to intersect the boundaries of the selected territories with gridded population data to estimate their population size in 2010 and 2020 (Appendix S4). Gridded population data allow for spatial and temporal comparisons and are used in conservation and development research (e.g., Newton et al., 2020; Venter et al., 2016) and other fields, including population and environment research, epidemiology, disaster risk reduction, and environmental monitoring. The suitability of particular gridded population datasets is context dependent, based on how well settlement patterns in a country or subnational region are captured by a product's (modeled) redistribution approach and the quality and spatial resolution of the input data (e.g., national census data or satellite imagery) (Fries et al., 2021; Hierink et al., 2022) (Section S1.2 in the Supporting Information Appendix). We evaluated four alternative 1×1 km datasets: gridded data (Grade Estatística) from the Brazilian Institute of Geography and Statistics (IBGE) (2010 data only) (https://geoftp.ibge.gov.br/recortes_para_fins_estatisticos/grade_estatistica/censo_2010/); 2010 and 2020 estimates from LandScan (https://landscan.ornl.gov/metadata); Gridded Population of the World (GPW) 4 (https://www.earthdata.nasa.gov/data/catalog/sedac‐ciesin‐sedac‐gpwv4‐apct‐wpp‐2015‐r11‐4.11); and WorldPop (their data product adjusted to match United Nations population estimates) (https://hub.worldpop.org/geodata/listing?id=75). Each product applies a different method to spatially redistribute Brazilian census data to pixel level and, with the exception of the IBGE Grade, predicting population change post‐2010.

We validated our territorial population estimates from the gridded datasets by comparing among them and with independent data sources, including all ILs (official Brazilian census data from 2010 and 2022 [https://www.ibge.gov.br/estatisticas/sociais/trabalho/22827‐censo‐demografico‐2022.html?edicao=37417&t=resultados]) separated by territory; all QTs (official Brazilian data from 2022 [https://www.ibge.gov.br/estatisticas/sociais/trabalho/22827‐censo‐demografico‐2022.html?edicao=37415&t=resultados]) aggregated by municipality (not territory specific); 25 RDSs and RESEXs (mostly in Amazonas) based on population counts in their management plans (median year of population surveys was 2011); seven RESEXs in Amazonas State based on 2007 IBGE population estimates (Dagnino, 2013); and complete population count from RDS Mamirauá in 2011 and 2019. WorldPop data provided the most reliable estimates of forest citizen population size. Compared with other gridded datasets, WorldPop‐based estimates were much closer to IL territory‐specific IBGE census counts (within 0.3% in 2010 and 0.4% in 2020 and 2022), RESEX and RDS population estimates from management plans (WorldPop = mean 15% lower), and Dagnino's (2013) estimates (WorldPop = mean 8% lower). For QTs, however, WorldPop‐based estimates were 24% below the 2022 IBGE census counts. Because WorldPop is derived from census data in which a person is only counted in their principal place of residence, our forest citizen estimates excluded seasonal residents of particular territories or those multisited residents that spend the majority of their time elsewhere.

We calculated municipality‐scale forest citizen intensity (FCI) as the proportion of the rural population constituted by the current number of permanent residents in the selected territories (Appendix S4). The municipal level represented the sum of the pixel‐level estimates within municipal boundaries.

## RESULTS

### Population size and distribution of forest citizens

**FIGURE 1:**
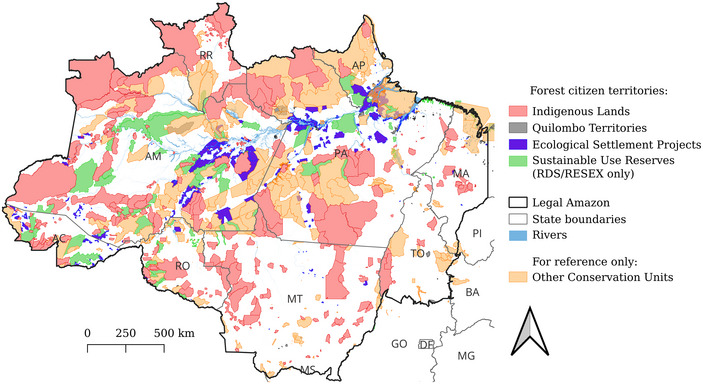
Forest citizen territories in the Brazilian Amazon (AC, Acre; AM, Amazonas; AP, Amapá; MT, Mato Grosso; PA, Pará; RR, Roraima; RO, Rondônia; TO, Tocantins, RDS, Reserva de Desenvolvimento Sustentável; RESEX, Reserva Extrativista).

We estimated that 1,054,102 forest dwellers inhabited 1411 Amazonian territories (across our four categories) that satisfied the preconditions for forest citizenship in 2020. The most populous territories were ILs (404,950 residents, 38% of all forest citizens), followed by ESPs (394,157 people, 37%), SURs (193,608 people, 18%), and QTs (61,387, 6%). ILs were home to the highest number of forest citizens in six of the nine Amazonian states, whereas SURs had the most forest citizens in Acre, ESPs had the most forest citizens in Pará, and QTs had the most forest citizens in Amapá (Appendix S5). Forest citizens’ territories collectively covered 1,585,738 km^2^, equivalent to 31% of the Brazilian Legal Amazon. The ILs were most numerous (613 territories), followed by ESPs (512 territories), QTs (151 territories), and SURs (135 territories) (Figure 1).

**FIGURE 2:**
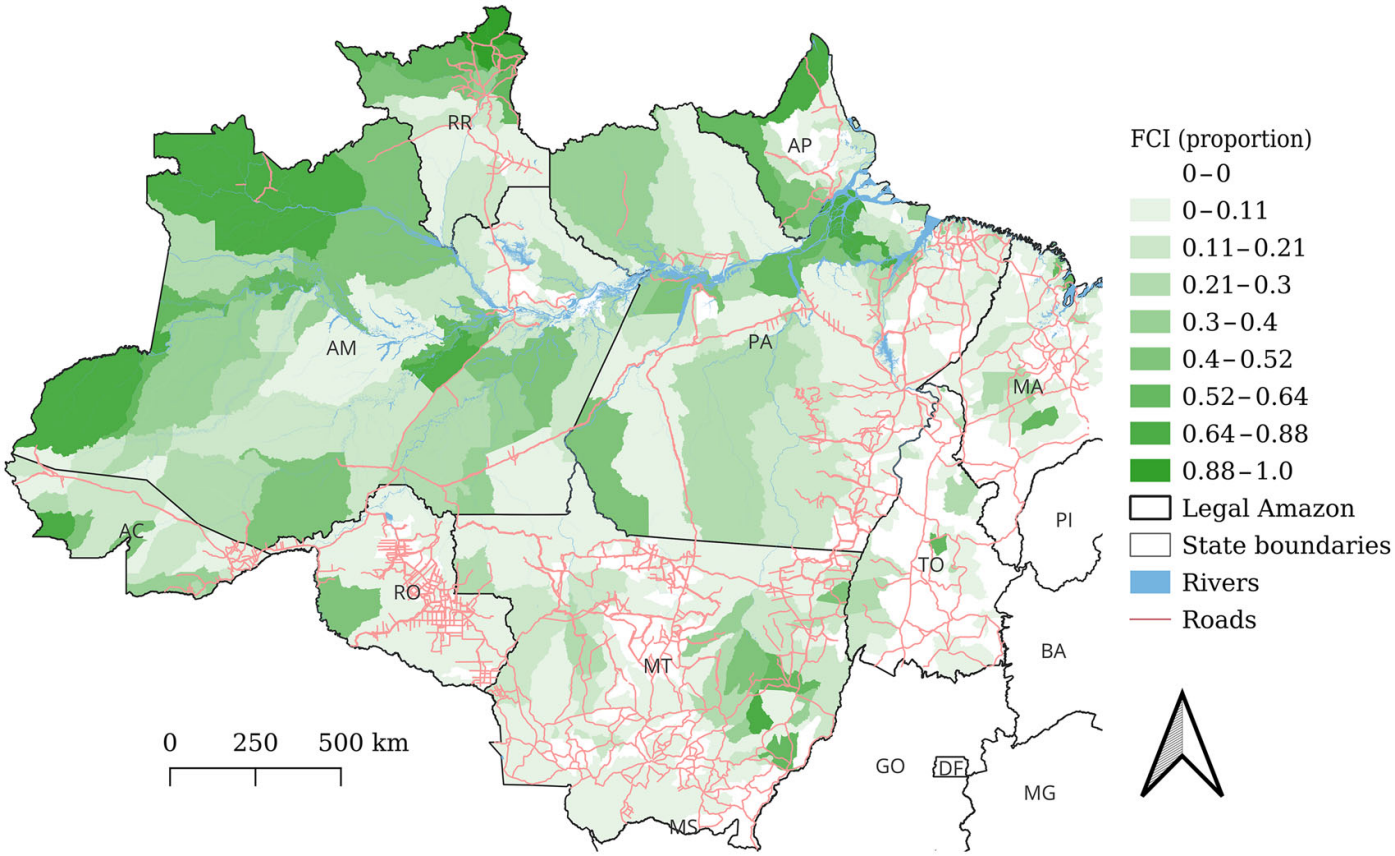
Map of forest citizen intensity (FCI) across the Brazilian Amazon, calculated as the proportion of a municipality's rural population in 2020 resident in forest citizen territories (AC, Acre; AM, Amazonas; AP, Amapá; MT, Mato Grosso; PA, Pará; RR, Roraima; RO, Rondônia; TO, Tocantins).

Forest citizens constituted 11.6% of the total rural population of 9,114,953 people (WorldPop estimate) in the Legal Amazon in 2020 (11.0% in 2010). Pará had the most forest citizens in 2020 (458,739 or 43.5% of the total), followed by Amazonas (267,853, 25.4%), Maranhão (105,428, 10.0%), Roraima (62,903, 6.0%), Acre (50,207, 4.8%), Mato Grosso (50,177, 4.8%), Amapá (30,136, 2.9%), Tocantins (14,902, 1.4%), and Rondônia (13,626, 1.3%). However, relative to the size of rural populations, municipality‐level FCI was highest in Roraima (mean = 0.39), followed by Amazonas (0.28), Acre (0.21), Amapá (0.18), Pará (0.12), Mato Grosso (0.06), Maranhão (0.03), Tocantins (0.02), and Rondônia (0.02) (Figure 2& Appendix S6). Across the Legal Amazon, mean municipal‐scale FCI was 0.09 in 2020, but it was highly variable (SD 0.16). Forty‐one percent (*n *= 317) of municipalities had zero FCI, whereas high FCI examples included São Gabriel da Cachoeira (Amazonas, 0.88), Marechal Thaumaturgo (Acre, 0.80), and Normandia (Roraima, 0.78). There was a negative correlation between FCI and the proportion of a municipality's remaining forest in 2001 that was lost by 2022 (Appendix S8). Some municipalities had high FCI and very low deforestation (e.g., Afuá on Ilha de Marajó), whereas others had moderate FCI and very high absolute deforestation (e.g., Lábrea, southern Amazonas). Many places with high deforestation had zero FCI (Appendix S7). The population in forest citizen territories grew by 174,801 people (19.9% increase) from 2010 to 2020; the greatest increases were in ILs (25% [SD 30]) (Appendix S15), followed by ESPs (19% increase [38]), SURs (15% [91]), and QTs (11% increase [44]). Territory‐specific IBGE census data for ILs showed 24% overall population growth from 2010 to 2022.

### Territorial creation processes, threats, and ongoing rights struggles

Formal steps in the creation processes varied across territorial categories (Section S9.2 in the Supporting Information Appendix). Bottom‐up mobilization to demand the creation of a particular territorial category was normally considered legitimate by the state only if voiced through a formal resident's association for a given area. Where an association did not exist, one had to be created. The impetus for proposing a territory or creating a resident's association sometimes involved external institutions as coprotagonists, but decision‐making remained exclusively within the communities. One resident or producer association sometimes succeeded in creating several territories. The process of creating these forest territories, and then implementing them, sometimes took many years.

Several land and natural resource threats to traditional communities emerged from our analysis. Major community concerns that motivated the creation of territories included wild‐cat gold mining and resource‐conflicts from outsider commercial harvesters taking, for example, fish, game, river turtles, trees, and Brazil nuts. Amazonian deforestation frontiers advanced through a mix of illegal logging, illegal land claims by *grileiros* (land‐grabbers) that were sometimes violent and were exacerbated by a lack of secure land tenure by traditional populations and related land speculation often based on clear‐cut deforestation to create cattle pastures. Additional concerns related to large‐scale projects included highway development and licensing of mining operations. A final motivation has been communities attempting to increase their visibility to the state in response to a perceived lack of public services or support for livelihoods in rural areas.

It was unclear how rights struggles within territories compared with the activism and experiences of forest communities without territorial recognition. However, literature and our fieldwork experiences highlighted six objectives of ongoing claims making by Amazonian communities, beyond their struggles for territorial recognition (Section S9.7 in the Supporting Information Appendix). The objectives overlapped between overcoming long‐standing barriers to rural people achieving rights and implementing the participatory management plan for communities in a forest citizen territory. A major objective was reducing obstacles to individuals being recognized as citizens by the state and included in official registers and being able to access public services and welfare programs. Barriers included lack of required documentation and difficulty in navigating complex urban‐centric bureaucratic processes. The latter was often compounded by costly trips from rural areas, absence of some government offices in some provincial towns, stigmatized treatment of forest dwellers by some public officials, low literacy, and administrative burdens imposed by public offices on low‐income citizens who cannot afford documentation.

Communities maintained their social and political organization to succeed with *associativismo* (community‐based organizing). People worked collectively and within democratic institutions to defend the interests of all residents within and across communities or those with shared social or livelihood identities. Community leaders negotiated with municipal governments to seek improved in situ access to public services. Such services included education, health care, social work, electricity, and clean water, including the employment of local people (e.g., as teachers), and acquiring resources for traveling to urban centers (e.g., means of transport, fuel). Communities attempted to overcome exploitation within economic structures to achieve fair access to markets and to improve incomes from rural livelihoods. Many bottom‐up associations—often with NGOs or government institutions—improved incomes by forming cooperatives, attempting to move up value chains (increasing market value by developing small‐scale processing, branding, and commercialization instead of only selling raw unprocessed materials), and developing innovative wildlife management projects. Communities attempted to protect local forests from land grabbing and deforestation by outsiders and to ensure sustainable resource use. Implementation of the forest territories placed environmental responsibilities on residents, including being vigilant against predatory resource extraction by outsiders and managing their own agreed on limits to their agricultural footprint, commercial selective logging, and wildlife harvest. In some territories, environmental stewardship was fostered by state institutions or NGOs. Finally, Amazonian communities attempted to cope with multiple kinds of shocks and stressors (e.g., rainfall and river‐level extremes, local‐scale river sedimentation or riverbank collapse, political shocks, epidemics of insect‐borne disease, and violence from drug trafficking and piracy).

## DISCUSSION

Forest citizenship has taken shape across the Brazilian Amazon through decades of struggles for territorial recognition. Working in equal partnership with forest citizens represents a socially just way of achieving Newing et al.’s (2023) call for people‐centered, rights‐based conservation. Our theorization of forest citizenship based on forest communities mobilizing to demand territorial recognition from the state and then claiming rights (e.g., to health, education) whilst attempting to conserve their forests represents a novel approach to understanding ecological citizenship in Brazil and potentially beyond. We combined the right to have rights—a cornerstone in Eurocentric political philosophy (see Lund & Eilenberg, 2017)—with Latin American thinking on ecological citizenship, which emphasizes confronting various forms of marginalization and injustice (Gudynas, 2009; Latta & Wittman, 2010), and two uniquely Brazilian influences. The latter include the fusion of social justice activism and discourse with environmentalism, which produced the policies and politics of socioambientalismo (de Castro, 2012) and Acre State's former political experiment with florestania, which attempted to extend citizenship to forest dwellers (Schmink, 2011). We calculated that 1.05 million forest citizens reside in territorial categories meeting democratic preconditions.

### Number of forest citizens

Amazonia's forest citizens constitute a large, culturally diverse group of people with a vitally important and outsized impact on conserving its ecosystems and biodiversity. We identified the territorial categories that are created through the bottom‐up demands of Amazonian communities and, later, have participatory governance. Forest citizens have unique relationships with these territories that encompass not only the physical space and forest visible in spatial analysis, but also social, legal, political, and economic dimensions (Aleixo & ATAMP, 2011). Our territorial selection of ILs, SURs, ESPs, and QTs is consistent with existing analyses of Amazonian sites of ecological citizenship (de Castro, 2012; Wittman, 2010). In Brazil legal recognition of territorial rights is the means by which laws and state institutions come to meaningfully acknowledge people's right to citizenship and land (Fraser, 2018) potentially overcoming differentiated citizenship (Holston, 2008). Territorial demands from below sometimes manifest through “autodemarcation” (acts of resistance and popular participation by IPLCs including the production of maps) (Vega et al., 2022). Territorial claims emerged in response to threats, including resource conflicts with outsiders (e.g., logging, fishing), wildcat gold mining, land grabs and deforestation, state abandonment, and state‐sanctioned megaprojects. Territorial boundaries reflect and shape the inhabitants’ identities, social relations, and political actions (Hecht, 2011; Little, 2003; Schmink, 2011). We showed how, during embryonic phases of territorial struggle, institutions, such as churches (Section S9.4 in the Supporting Information Appendix) and NGOs, may serve as coprotagonists with Amazonian communities to strengthen communities’ political organization, but decision‐making power must remain with communities. Hence, forest citizenship should not empower NGOs to talk on behalf of forest dwellers, which would be undemocratic (Pieck, 2013). In many territories, elected resident associations play central roles in forming councils and developing and implementing management plans. These institutional structures have democratized conservation and natural resource management (Pickering et al., 2020) in the Brazilian Amazon.

Democratic environmental governance involved forest‐proximate people in several territorial categories. Foremost, ILs were home to 38% of all forest citizens, but ESPs (37%), SURs (18%), and QTs (6%) were also important. The relative importance of different territories varied in space; in Pará most forest citizens lived in ESPs. Creation of forest territories slowed markedly after 2010 (Gomes et al., 2018) (Appendix S5), yet we found their total resident population grew 20% in the decade to 2020. This likely exceeds the potential natural rate of increase and may reflect forest dwellers returning from urban areas to their communities of origin (H.C. Pereira, personal communication, 2023). The creation of 1411 territories covering 31% of the Legal Amazon is emblematic of enormous collective success of bottom‐up mobilization by Amazonian communities (de Castro, 2012), yet only 12% of rural people in the Brazilian Amazon live in conditions foundational to forest citizenship. Understanding spatiotemporal variation in FCI could be useful to researchers studying diverse kinds of environmental outcomes or human welfare issues and to decision makers deciding where to prioritize or target funding, projects, or policies.

### Practices of forest citizenship

Forest citizenship becomes tangible through rights claims of Amazonian communities that have achieved territorial recognition (i.e., nascent forest citizens). We argue that Amazonian communities must work collectively to overcome challenges, including precarious provision of education (Pereira et al., 2022) and health care (Garnelo et al., 2020), unfavorable access to markets and trade (Section S9.7 in the Supporting Information Appendix), resisting violent resource grabs by outsiders (Lobo & Cardoso, 2023; Nepomuceno et al., 2019), climatic change (Chacon‐Montalvan et al., 2021), drug trafficking, and piracy (IPEA, 2023). To address these challenges, Amazonian community leaders seek, attract, and maintain relationships within communities, and with other communities and external institutions (Mathews, 2021). Rural internet access allows for new forms of engagement and, potentially, claims making (processes of communities articulating their demands in political or public arenas). For example, Ribeirinhas da Amazonia, a youth‐led YouTube channel based in RDS Amanã, has >135 million views and 420,000 subscribers. Can successful strategies and tactics of forest citizenship (following Shankland, 2010) from particular territories be nourished elsewhere? Ultimately, understanding practices of forest citizenship requires in‐depth, mixed methods research in diverse territorial contexts. Research should establish whether the rights claims of forest citizens are more extensive, organized, or successful than those of other rural communities.

Forest citizenship needs to be substantive (Chigudu, 2019) and lead to tangible improvements in people's lives, including through greater access to high‐quality public services. On the Rio Juruá, livelihoods, access to basic services, and infrastructure are all better inside the SURs than outside (Campos‐Silva et al., 2021). To fulfill the democratic promise of territory‐based forest citizenship, benefits should emerge through enhanced agency and participation, though the latter can be tokenistic. Preliminary findings from our qualitative fieldwork showed that forest citizens face many state‐created burdens in accessing public services. These burdens are related to, for example, documentation, transportation, language, and practices of bureaucrats disconnected from rural realities. Moreover, the collectivity central to the creation and management of these territories places some limits on the freedoms of individual forest citizens. A forest citizen's right to territorial security depends on the strength and unity of the citizen's resident association. For example, a single resident in a SUR cannot, alone, gain the right to commercially harvest pirarucu (*Arapaima* spp.). Forest citizens’ realistic opportunities for achieving their rights appear to depend mostly on community‐level claims and less so on individuals. Is forest citizenship therefore best conceived as a form of collective ecological citizenship?

### Subcitizenship in Brazil

Currently, it is unclear which conditions are necessary for forest citizenship to be emancipatory and which turn it into a new form of domination—both could be present even within a single territory. Souza (2003) explored the concept of subcitizenship in Brazil, emphasizing how the normative idea of citizenship is often publicly asserted yet denied in everyday life through diverse forms of violence and segregation. Influenced by Fernandes (e.g., 1975), Souza (2003) asserts that Brazil retains deep social divisions rooted in centuries of slavery. A social structure that has not changed its basic form since the colonial period will clearly be a barrier to full realization of citizenship. In modern societies, citizenship rests on states recognizing individuals’ rights and distinguishing social groups that have or lack access to rights and guarantees. Construction of the citizen as a subject of rights and duties is only possible in the legal space defined by the state. Castro‐Gomez (2011) posits that the state fulfills its judicial‐political function of inventing citizenship by setting criteria and boundaries for qualification. The resulting identity categories enable a state to exercise control over a population. Discrimination against different citizens reflects societal marginalization and is perhaps inherent to modern government. In Latin American states, citizenship was built historically on hegemonic ethnic identity, which violates the physical and moral existence of Indigenous and Black communities. Despite the progressive 1988 Constitution, Brazil's authoritarian past has had lasting effects on Amazonian peoples and resulted in “differentiated citizenship” (Holston, 2008).

How realistic, then, is the image of forest citizens claiming rights by holding public officials to account given the country's high levels of state‐sanctioned violence and shortcomings in the democratic rule of law (Pereira, 2020)? For Souza (2009), the exploitation of Brazil's subcitizens takes economic and symbolic forms, including the function of humiliation in daily life, eroding self‐confidence, and reactive capacity. The effects of institutional and social stigmatization on people's sense of self‐worth are well demonstrated by research on health care access among Brazilian street dwellers (Teixeira et al. 2019; de Queiroz et al., 2022). Souza (2009) drew on Bourdieu's (2007) work on social distinction to argue that subcitizenship is often perpetuated through unconscious prejudice around skin color, clothing, accent, language, or tastes in music or food. The stigmatization and humiliation of rural Amazonians in their interactions with public institutions is commonplace (e.g., Nepomuceno et al., 2019:129–130), and the continued dominance of historical elites in provincial Amazonian municipalities (Abel, 2022) necessitates reconfiguration of social and political relations beyond territorial boundaries.

### Limits to forest citizenship

There is a tension between citizenship's inclusionary promise and exclusionary tendency (Kabeer, 2005). As Fraser (2018:729) observed when comparing struggles for recognition on the Madeira and Tapajos Rivers “the state emerges as complex and Janus‐faced [duplicitous]: its institutions can facilitate as well as be an obstacle to emancipatory struggles.” This is partly because inclusion through participation in spaces provided by the state can create differentiated access, for example, where forest dwellers face long journeys to reach government buildings. Not everyone may speak the political language, including acronyms used by public servants (Cornwall & Shankland, 2013). Participation may harm participants (de Souza Santos 2019), and—as territorial advocates—community leaders have been targets of extreme violence. Using territorial boundaries to define forest citizens may also create a “dynamic of exclusion” (Staeheli 2010) in relation to other forest dwellers. Dialogue on forest citizenship should avoid inadvertently portraying the millions of rural Amazonians living outside particular territories (ILs, QTs, SURs, ESPs) as noncitizens who are somehow less deserving of rights. Bryan (2012) contends that territory makes space governable through the recognition of rights and distribution of political authority and that rights make people governable.

Forest citizenship will only achieve its transformative potential by being insurgent and empowering marginalized people (Holston, 2008). Citizenship from below challenges citizenship's exclusionary tendency and emphasizes legal recognition (e.g., of land rights), intersubjective recognition (mutual acknowledgment of existence and worth) (Vega et al., 2022), and self‐determination, justice, and solidarity (Lister 2007). Nonetheless, forest citizenship might be coopted by the state, capital, and large NGOs given that it involves state‐sponsored forms of participatory governance (Latta & Wittman, 2010, de Souza Santos 2019). In Peru, for instance, ecological citizenship channels protest and citizen participation into recognized institutional forms subject to rules laid down by the state (Pieck, 2013). Moreover, the collective actions of forest citizens should not replace the state's own duties to its populace, and they should not bear the significant costs of managing and defending Amazonian forest territories (Brondizio et al., 2009). Although forest citizen territories have participatory governance, rural Amazonians’ livelihood opportunities remain influenced by government regulations on the circumstances in which harvesting particular natural resources is considered sustainable and permissible (Antunes et al., 2019). Together with economic boom‐bust cycles and changing priorities of the institutions financing Amazonian conservation, environmental regulations have compelled forest citizens and other forest dwellers to adapt their livelihoods.

### Forest citizens elsewhere

To our knowledge, no countries have replicated Brazil's post‐1988 policies of creating particular kinds of common‐use territories for marginalized populations. Yet, fostering territory‐based forest citizenship could benefit people living elsewhere, especially in Latin American countries where post‐1970s environmentalism has been linked to struggles for democracy, including community land rights (Latta & Wittman, 2012). Brazil has much in common with its neighbors, including multiculturalism, a colonial history of oppression and slavery, emergence from authoritarianism in the 1980s, and social and environmental concerns about development centered on export of agricultural products and natural resources (Latta & Wittman, 2010). The role of territorial rights in Brazil's social‐environmental history and class struggle has similarities with Mexico's rights recognition of forest peasants (Kashwan, 2017). For example, Brazil’s Extractivist Reserves (originally, rubber‐tappers fighting dispossession by cattle‐ranchers) and agrarian reform settlements (reflecting bottom‐up activism against landlessness).

Our conceptualization of forest citizenship could be applied universally given that it draws on Arendt's (1951) internationally well‐established political theory and is coherent with a classic perspective of rights‐based citizenship. Intriguingly, in 2019 at least 313 million people lived within 1 km of a forest in low‐ and middle‐income countries that were liberal democracies (defined as >0.40 on the V‐Dem index from low to high [0‐1], calculated based on Global Change Data Lab, [2023] and Newton et al., [2022]). Yet, forest citizenship's relevance is contingent on territorial arrangements; national, colonial, and postcolonial histories; democratic structures; land rights; and contemporary political circumstances. The kinds of legally recognized forest territories in a country reflect the spatial distribution of cultural groups, such as Amerindian populations, Afro‐descendants of enslaved people who resisted slavery, traditional forest extractivists, and landless people. Colonial legacies also influence whether forest dwellers can obtain land tenure or use rights. For instance, Mexico's communal lands reflect Castilian philosophy of social production through common land ownership, contrasting with India and Tanzania's tendency for fortress conservation, following a British preoccupation with land's economic productivity (Kaskwan, 2017).

### Policy implications for Brazilian people‐centered conservation

The efforts and successes of forest citizens in protecting their forests and fauna (e.g., de Assis Barros 2022; Campos‐Silva et al., 2018) appear to show that citizenship as a democratic practice can coexist with another normative framing, sustainability (Pickering et al., 2020). Forest citizens have demonstrated a strong commitment to their territories through long‐term investments of time, political energy, and other resources into livelihoods‐based sustainability initiatives in their forests, lakes, and rivers. This is not the case for all Amazonian rural populations. People‐centered conservation, however, must focus on power relations and decision‐making structures as much as on conservation outcomes. If it is indeed transformative, forest citizens should already have reconfigured their social relationships with NGOs, researchers, state institutions, and other outsiders and upended hierarchies in which they had frequently been subordinate. Echoing praise of ecological citizenship in Latin America (Oliveira, 2014), we believe forest citizenship helps avoid two false, unhelpful characterizations of Amazonian forest dwellers as “agents of degradation” or “docile stewards of nature.” Conservationists must recognize and support the diversity of these social groups and their territories.

Achieving people‐centered conservation requires that conservationists respect the rights of forest‐proximate peoples (Newing et al., 2023). Implementation of this under the Kunming‐Montreal Global Biodiversity framework may benefit from drawing on key insights from decades of social science scholarship. First, conservation initiatives in the Global South must ensure genuine participation (see Cooke & Kothari, 2001) and recognition at all stages (Martin et al., 2016). As Fleischman & Solorzano (2018) show for India and Mexico, oppression of the rural poor in postcolonial states can hamper meaningful participation in conservation projects. Second, confronting marginalization and rights violations—including of forest dwellers—means tackling imbalances in the exercise and distribution of power. Achieving rights‐based conservation requires looking beyond ecological sustainability and communities’ material economic needs and engaging with the social and political dimensions of forests (Hecht, 2011; Vandergeest & Peluso, 2015). Third, some conservation projects can, inadvertently, undermine democracy and reproduce social marginalization. For example, some REDD+ projects commodify benefits from nature and recruit Indigenous people to be unwitting custodians of carbon credits (Latta & Wittman, 2012). Fourth, history matters. In many regions of high conservation interest, the perceptions, attitudes, and wariness that rural people may have of state institutions and international actors is shaped by experiences of state‐sanctioned violence (Nixon, 2011). Such violence ranges from enslavement, colonial exploitation, and forced migration to more recent dispossession, police violence, and premature death through ill health and disease.

Our conceptualization of forest citizenship emphasizes access to rights and democratizing conservation in Amazonia, rather than the environmental duties and responsibilities associated with ecological citizenship in the Global North (Pickering et al., 2020; MacGregor, 2014). The existence and visibility of the institutions in identified territories provide easy points of entry for external actors and agendas, raising opportunities and risks for forest citizenship's emancipatory potential. Risks include green washing and carbon finance schemes that may provide forest dwellers with “precarious inclusion” (Greenleaf, 2021) and accrue benefits to intermediaries. Conservation science and practice can have profound impacts on how forest peoples see themselves, and how they relate to and use nature. Agrawal (2005) showed how forest dwellers can become environmental subjects. Amazonian territories risk being treated as laboratories in which NGOs and researchers can experiment with behavioral change techniques to alter forest dwellers’ environmental attitudes and behaviors. Merely working with forest dwellers does not mean that a conservation intervention is necessarily democratic or just.

### Study Limitations

Several limitations in our study reinforce the need for more conceptual and empirical research on this topic. Citizenship is present in some bottom‐up discourse (e.g., Aleixo & ATAMP 2011), but does forest citizenship resonate with Amazonian people's own interpretation of their activism and struggles for territory and other rights? We are receptive to disagreements about our choices of forest citizen territories and highlight that relatively little research has been conducted in ESPs or QTs. Perhaps we overestimated participatory governance in some categories or erroneously concluded that forest citizenship is not fostered in others. The prospect of forest citizens emerging through novel kinds of territory (e.g., *territórios de uso comum* [common use territories]) is exciting. The unavailability of territorial polygons of the SUR and ESP territories not yet fully created means we may have overlooked the bottom‐up practices of citizenship playing out in those places. Future researchers could refine our binary classification based on category and analyze democratic processes in specific territories (e.g., having an approved management plan). Our assumption that forest citizens live all or most of the time in their rural homes is contestable given that decades of rural‐urban migration in Amazonia have resulted in complex, multisited lives and livelihoods for many people. Cases of seasonal resource users or household groups moving between rural and urban locations add a shade of gray to the territory‐based conception of forest citizenship. Finally, we lacked a consistent source of recent population data, pending future availability of gridded data from IBGE's 2022 census.

Forest citizens are a large, diverse group of people with outsized conservation importance due to their commitment to protecting forests and sustainably harvesting natural resources. Environmental governance in their territories must be democratic, rights based, and genuinely participatory. People‐centered conservation means conservationists need to avoid false and simplistic characterizations of forest citizens and recognize the diversity of these social groups and their territories. We positioned territorial recognition and the right to have rights as a profoundly important democratic achievement in the Brazilian Amazon and the basis of forest citizenship. The bottom‐up territorial struggles of peasant and Afro‐indigenous movements, often in partnership with environmentalists, have created a socioenvironmental frontier in Amazonia, slowing forest loss and degradation (Domingues & Sauer 2023). We argue that forest citizenship in Amazonia—as a normative framework and way of understanding what is already happening—should emphasize rights, not environmental responsibilities, given the historical marginalization, oppression, and exploitation of Indigenous and traditional populations. Forest citizenship is therefore a distinctly Latin American form of ecological citizenship, where rights struggles for marginalized rural people are fundamentally linked to struggles for territory and livelihoods (Latta & Wittman, 2012). Some skepticism is needed about the concept of citizenship in highly unequal contexts (Latta & Wittman, 2012), and forest citizenship's emancipatory, radical potential is contingent on its ability to tackle entrenched inequalities. Beyond Brazil, our theorization and population estimates of a particular kind of ecological citizen contributes to work on democratizing environmental and natural resource management, the so‐called democracy‐environment nexus (Pickering et al., 2020).

## Supporting information



Supplementary Materials.
