# Supplementary material for: Forest citizens and people‐centered conservation in the Brazilian Amazon
Source: Conserv Biol. 2025 May 30;39(3):e70031. doi: 10.1111/cobi.70031 (PMC12124167; doi:10.1111/cobi.70031)
Supplement: Supplementary file 1 — Supplementary Materials. [file COBI-39-e70031-s001.pdf]

Supporting Information

Appendix S1. Territorial categories meeting criteria as foundational for forest citizenship

1.1 Included territorial categories

Based on the Methods described in the main paper, including collaborative discussions at two workshops in Brazil in 2022, we determined that the available evidence indicates the following territorial categories satisfied – in general – the two democratic preconditions (community mobilization in the creation process, and later having some level of participatory governance) and attempts to conserve forests. This environmental condition is principally based on the presence of an environmental agenda (and forest protection goals) in the legislation related to particular territorial categories. The alternative, using data on avoided deforestation related to territorial creation, is challenging because of (a) the empirical difficulties in demonstrating causality given the spatial factors which confound the creation of protected areas and related measures of their effectiveness, (b) many Amazonian territories in the identified forest citizenship categories are far from deforestation frontiers (Joppa & Pfaff 2009; Kere et al. 2017), and their environmental goals go beyond avoiding clearcut deforestation. For instance, the conservation of a flooded forest ecosystem may relate more to avoiding commercial overfishing, selective logging, and gold-mining presence, as opposed to more-easily detectable effects on deforestation rates.

We define the following territories as fulfilling these preconditions for forest citizenship:

(i) **RESEX and RDS sustainable use reserves (SURs)**. These are protected areas, located in forested landscapes, which prohibit or constrain intensive usage of natural resources. Only two categories were considered, extractive and sustainable use units. Federal RESEXs and RDSs are created and comanaged by the federal Chico Mendes Institute for Biodiversity Conservation (ICMBio), within the Ministry of Environment. State-level RESEXs and RDSs fall

within State Environmental Secretariats (known as SEMA).
(ii) **Indigenous lands (ILs):** also protected areas, but reserved for Brazilian Indigenous populations. They are created through a legal-administrative process involving multiple levels of federal government and indemnification (compensation for loss or harm) of non-indigenous residents. ILs are under the jurisdiction of FUNAI, within the federal Ministry of Indigenous Peoples.
(iii) **Quilombola territories (QTs):** reserved for the descendants of African people enslaved in Brazil in the XVI and XIX centuries. Their creation also entails a multi-stage legal-administrative process. QTs are under the authority of the National Institute for Colonization and Agrarian Reform (INCRA), a selfgoverning unit within the Ministry of Agriculture. However, QTs can also be created by other state-specific land institutions and recognition of Quilombola territorial claims is in conjunction with the state-affiliated Palmares Cultural Foundation.
(iv) **Ecological settlement projects** (our term; ESPs) of three sub-categories (*PAE, PAF, PDS*), which in Portuguese are collectively known as *Projetos de Assentamento Ambientalmente Diferenciados* (Environmentally-differentiated Settlement Projects). These are created as part of agrarian reform, generally targeting agricultural use of land. The types selected have special goals related with sustainable resource management, especially forest resources. In other words, they are *intended* to simultaneously achieve social justice, smallholder land management, and environmental conservation (Porro & Porro 2022). ESPs are also under INCRA authority, managed by various regional superintendencies. Relevant to ESPs, Wittman (2010) analyzed ecological land reform in Brazil as part of the 2003 federal government's agenda of supporting the "*extension of democratic citizenship to the rural population*".

Our list resonates with de Castro's (2012) work on territorial sites of ecological citizenship (albeit he omitted RDSs); these categories have some official emphasis on social justice, land rights, rural development, and forest conservation. This emphasis partly reflects the goals and political motivations of the relevant government ministry.

1.2 When included territories become meaningful for rights-claims

Legal frameworks shape the stage at which the different kinds of territories become effective in achieving particular community goals (e.g., to contest plans to allow commercial mining near to a community). We considered IL legally-meaningful from when they first become spatialized (boundaries represented by polygons in a government shapefile) during the 'under study' early stage in the lengthy creation process, because Indigenous communities have successfully won court cases based on using these boundaries to represent their land-rights. We included QTs from preliminary demarcation (based on on-site anthropological and socioeconomic study) due to community success in drawing on them in land-disputes (Benzeev 2022).

We did not include the first 'declaration' stages of QTs and ILs because a lack of agreed spatial boundaries means, (a) we are unable to calculate the resident human population, (b) a community/ies would likely fail to defend their land-rights in court, and (c) For ILs, this very early stage in the land-tenure process does not protect against deforestation (Benveez et al 2023). For SURs and ESPs we were restricted to fullycreated territories (Phase 3 and higher for ESPs) due to the lack of reliable shapefiles for earlier stages in the creation process.

1.3 Excluded territories

We consider that tolerance is imbued with disapproval and regulation therefore incompatible with the emancipatory promise of forest citizenship. Our aversion to tolerance is based on Brown's (2006) seminal book, and author M.T.'s experience of land conflicts in Amazonia. According to Brown, tolerance is not to affirm but to conditionally allow what is unwanted or deviant, and can involve consolidating the dominance of the powerful. Interpreted by Staeheli (2010), tolerance, "*seems like a universal value that should be hard to contest, but its apparent universalism and neutrality masks the ideological work it does in designating only certain practices and certain ways of being as appropriate to citizens.*".

In addition to *Florestas* (Forests), other categories of Sustainable Use protected areas (as defined by the Brazilian government) which did not fulfill the criteria of

bottom-up mobilization in creation, or participatory governance included *Área de Proteção Ambiental* (APA), *Área de Relevante Interesse Ecológico* (ARIE), *Reserva de Fauna* (REFAU), or *Reserva Particular do Patrimônio Natural* (RPPN). Traditional riverine communities are also tolerated within some National Parks (e.g., P.N. Jau in Amazonas), especially when the communities existed prior to the territory's creation.

De Castro (2012) included all types of agrarian reform settlement, whereas we only consider the ESPs, given higher rates of deforestation in conventional *assentamentos* (Pereira et al. 2022), including increasing land concentration in some (i.e. reversing agrarian reform; Yanai et al. 2020). Nonetheless, Wittman (2010) showed even within a conventional *assentamento* in Mato Grosso State, rural people with collective land tenure can work successfully to minimize illegal logging, deforestation and forest degradation by outsiders.

Appendix S2. Quantifying forest citizens in the Brazilian Amazon

2.1 Gridded population products

We used gridded data because territory-specific official universal population counts were only available for ILs, and Brazilian official census sectors tend to change between censuses, and do not map well onto the boundaries of our selected forest territories. The reliability of gridded population data is context-dependent. We used: (i) the official gridded dataset (*Grade Estatística*) from the demographic census of the Brazilian Institute of Geography and Statistics (IBGE; 2010 data only); and 2010 and 2020 estimates from (ii) LandScan from Oak Ridge National Laboratory, USA; (iii) Gridded Population of the World (GPW) v4 from NASA, USA; (iv) WorldPop (UN adjusted) from the University of Southampton, UK.

In a global study of urbanization trends, WorldPop data was closer to UN projections than Landscan or GPW4 (Hanberry 2022). A Sub-Saharan African study reported large differences in health access based on different population datasets, concluding that WorldPop constrained was most reliable for that context (Hierink et al. 2022). In a mountainous region in Southwest China, WorldPop performed better than GPW or

LandScan when validated against local population data and GoogleEarth imagery (Xu et al. 2020). However, in Bioko Island, Equatorial Guinea, LandScan was most accurate for urban populations, unconstrained WorldPop and GPS were relatively poor in all contexts, whereas constrained WorldPop matched well in low density rural areas (Fries et al. 2021). Some products constrain population to cells with detected buildings (e.g., LandScan, WorldPop top-down constrained, High Resolution Settlement Layer [HRSL]), whereas others do not (e.g. WorldPop unconstrained). Population estimates based on a single population dataset (e.g. Newton et al [2020] used LandScan data to calculate a global total of 1.6 billion rural forest-proximate people in 2012) are subject to unknown methodological limitations and biases.

2.2. Details of candidate gridded population datasets

Given we are not aware of any study validating the accuracy of gridded population data for the Brazilian Amazon, we evaluated four gridded population datasets (Appendix S7, S8). The IBGE grid product applies a hybrid approach to estimating populations, combining household GPS coordinates from the 2010 (where census agents were able to obtain them) with a redistribution approach for those households without coordinates, within a given census sector. This redistribution method was constrained by the kinds of gridded land-data available during the development of the grid prior to release in 2016 (reliant on TerraClass, rather than the superior MapBiomas data which is now the gold standard for environmental research in Brazil)(H. Pereira, personal communication). IBGE recognizes a negligible level of under-counting with the gridded product, compared to sector-level information (IBGE 2016), but the under-counting may be spatially variable. IBGE documentation implies redistribution was based on the road network, perhaps making it less suitable for riverine populations in Amazonia.

The GPW dataset assumes even population distribution within census administrative boundaries, after excluding water bodies, and does not use secondary data in its modeling (NASA, 2018). This lack of spatial precision is a limitation but also means this data can be combined with other variables without the risk that they are endogenous to the population estimate (Bustos et al 2020). Artificial intelligence, more specifically, machine learning, was incorporated into the algorithms of Landscan and WorldPop.

LandScan data is highly modeled using proprietary algorithms, without much transparency about the process. Constant refinements of the modeling process limit the reliability of time-series comparisons (Bustos et al 2020). WorldPop data is derived from Random Forest Modeling which predicts population density using available census data – partially based on GPWv4 – and covariates (which may vary by country) including land cover, elevation, urban expansion, nighttime lights, schools, health clinics (see Stevens et al. 2015). Worldpop also utilizes demographic spanning based on pregnancies, births, and migration (WorldPop, 2024). Higher resolution (approximately 100m squared) WorldPop data is available for Latin America (Sorichetta et al. 2015).

2.3. Independent sources for validating gridded population datasets

Our validation sources included (a) all ILs (official Brazilian census data from 2010 and 2022) separated by territory; (b) all QTs (official Brazilian data from 2022) aggregated by municipality, not territory-specific. The resident population of QTs was not available from the Brazilian 2010 Census and hence we could not calculate census-based growth rate for QT; (c) 25 RDSs and RESEXs (nearly all in Amazonas state) based on population counts in their official Management Plans around 10 years ago; (d) Dagnino et al. (2013)(7 RESEXs in Amazonas State, 2013, based on official IBGE population estimates in 2007); (e) a complete population count from the RDS Mamiraua, Amazonas in 2011 and 2019 by the Instituto Mamiraua.

Our WorldPop-based calculation of the total 2020 forest citizen population (i.e. all people living in selected territories) was 39% higher than Landscan and 45% higher than the GPW-based estimates. WorldPop data was closer to Management Plan population counts for selected RESEXs/RDSs ([c], above) being 15% below, on average. Relative to the Management Plan data, the gridded 2010 IBGE data was 22% lower, on average, LandScan data was 97% above, and GPW data was 80% below. Relative to Dagnino's population estimates for RESEX, WorldPop was 8% lower, on average, whereas other data sources had much higher deviance (Appendix S7,S8). Census data from the Instituto Mamirauá shows the population of the RDS Mamirauá grew 7.7% between 2011 to 2019 from 5,780 to 6,224 people, whereas, based on

WorldPop, the most accurate gridded population source, the estimated population change was 18% decline from 6,221 in 2010, to 5,122 in 2020 (Appendix S10). Regarding population growth rates for two other reserves with Management Plan population counts across two years, RDS Rio Amapá experienced 35% total population growth 2010-2018 compared to 10% growth using WorldPop (2010-2020), and RDS Uatumã had 25% growth 2007-2016, compared to 36% using Worldpop (2010-2020).

2.4. Exclusion of occasional residents from our estimates

Note that population data sources (c) and (e) will often include multi-local households and individuals who are *sometimes* resident in a particular territory. In contrast, governmental demographic censuses strive to avoid double-counting and hence would count someone only in either a rural community or, for example, a nearby town. To reiterate, WorldPop data is derived from a country's official census data, and remodeled to account for fine-scale population distributions (within census sectors) and population change over time. In Brazil, IBGE census agents seek to establish who was resident in a household on a particular reference date, and follow guidelines for situations in which a person or a household splits their time between two or more locations (e.g. an urban area and rural community) (IBGE 2010, p53). The principal residence is indicated by the interviewee. This is clarified by the census agent by asking about the location in which the interviewee spends most of their time during the course of the year. If split evenly, the principal residence is the home in which the interviewee has lived for longer. If no-one in a household considers the home their principal residence it would become classified as Household of Occasional Use (which would return a zero in the population count). A person is therefore only counted as a resident in the place in which they normally live.

Many former residents of Amazonia's remote rural areas now reside in nearby urban centers but may continue to spend time in rural areas for small-scale farming or harvesting natural resources (Parry et al. 2014). For example, some former residents of a given territory may spend weeks or months there each year during the Brazil nut harvesting season, or participate in community fishery management alongside their kin and peers. Other multi-sited people or households may spend the majority of time

somewhere else. For instance, someone may work or study in a nearby town during the week, and spend their weekend in a RESEX reserve (e.g. see Dodd 2020). Because our population estimates of forest citizens consider only permanent residents of a territory, and exclude people who spend most of their time elsewhere, they should be taken as conservative minimums. For this reason, territorial population counts from (c) and (e) are likely to be higher compared to sources directly or indirectly based on the governmental IBGE census (i-iv; and a/b). Part-time or seasonal residents may still engage in practices of forest citizenship either through their livelihoods and related institutional structures (e.g. fisheries management of lakes) or perceive themselves part of the territory due to their involvement (whether previous or on-going) in social movements.

2.5. Census of resident population

Our novel population estimates and the validation data we utilized all related to the permanent resident population within selected territories (see Appendix S2 for examples). Consequently, we count all permanent residents within ILs and QTs rather than only households which self-identify as indigenous or Quilombola. By focusing on residents, we exclude communities in the buffer zones (*zona de amortecimento*) of RDS and RESEX territories which are normally considered resource-users (*usuários*) in Management Plans. *Usuário*s in buffer zones are normally subject to norms and legal restrictions intended to minimize negative impacts of resource use on the protected area, clarified in the Management Plan and state or federal law (e.g. in Amazonas there is the SEUC - Sistema Estadual de Unidades de Conservacao). In this sense, *usuários* may experience a form of ‘differentiated’ forest citizenship where they are subject to duties and environmental responsibilities but denied access to some rights. In practical terms, buffer zone communities may benefit from the productivity benefits and market access afforded by participating in collective management of fisheries, for example. Note that neighboring *usuários* of a given territory may be resident in another qualifying territory. In many cases, communities of *usuários* living in a territory’s buffer zone would have previously chosen not to mobilize or participate in the collective process of creating a particular kind of territory.

2.6 Calculation for estimating forest citizen populations

The population inhabiting the i-th target territory, i.e., the forest citizen population located in the i-th territory, FCi, was calculated as a weighted total. The population recorded in the j-th grid pixel was equitably assigned, based on overlapping land area, to all territorial units which intersected the j-th pixel. That is:

$$FC_i = \sum_{j=1}^{N} w_{i,j} FC_j = \sum_{j=1}^{N} \frac{a_{i,j}}{A_j} FC_j$$

In which wi,j is the weight of the j-th cell in the i-th territorial unit, which equals the share of the cell's area intersecting the i-th unit, i.e., the ratio of overlapping area (ai,j) and cell's area (Aj). The weights are multiplied by the forest citizen population of the j-th cell (for instance, if a pixel is intersected in 30% of its area by a unit, then 30% of its population is attached to the unit).

The weights do not sum to one within a given territorial unit (only across all overlaps of a given pixel). Hence, rigorously, the calculation is a "weighted total" instead of a weighted average, i.e., it informs the total population at the level of the whole unit and not the average at the level of pixels.

2.6. Territories considered: sources and description

Digital maps showing the boundaries of four special territories, owned by the Brazilian government, were merged. Seeking to avoid double counting, overlaps of units belonging to different territories were identified and assigned randomly to only one territory. See Appendix S1.

Appendix S3. Characteristics of source datasets from territories classified as meeting preconditions for forest citizenship

Territory	Source	Included types & source website	Included phases	Ref. date
Selected conservation units (RDS, RESEX)	Brazilian Ministry of Environment	Extractive and sustainable use reserves (direct resource use allowed) only. Federal, state and municipal http://mapas.mma.gov.br/i3geo/datadownload.htm	Only units with fully completed official recognition process were available in the original shapefile	Aug 2022
Indigenous lands	Brazilian National Indigenous Foundation (FUNAI)	N/A/ (no sub-categories) https://geoserver.funai.gov.br/geoserver/web/?2	All phases of the official recognition process: 1, under study ("em estudo"); 2, delimited ("delimitada"); 3, recognized by the Ministry of Justice ("declarada"), 4, recognized by the Brazilian presidency ("homologada"); 5, recognized by notary office as governmental property ("regularizada"). Also included donated land, (doesn't go through the process). Known as "encaminhada como reserva indígena").	Mar 2023
Quilombola Territories	Brazilian National Institute of Colonization & Agrarian Reform (INCRA)	N/A/ (no sub-categories) https://acervofundiario.incra.gov.br/i3geo/datadownload.htm	All phases of official recognition process: 1, technical report defining area's boundaries ("elaboração do RTID"); 2, recognition by INCRA ("portaria"); 3, presidential decree mandating dissappropriation of previous residents ("decreto"); 4, partial land title granting ("titulação parcial"); 5, final land title granting ("titulação"). Also included unknown phase "NA"	Aug 2022
Ecological Settlement Projects	INCRA	PAE, PAF, PDS (particular kinds of 'environmentally-differentiated' Agrarian Reform Settlements) https://acervofundiario.incra.gov.br/i3geo/datadownload.htm	Only those that were officially created (known as "phase 3", or higher) were available in the original shapefile.	Sept 2022

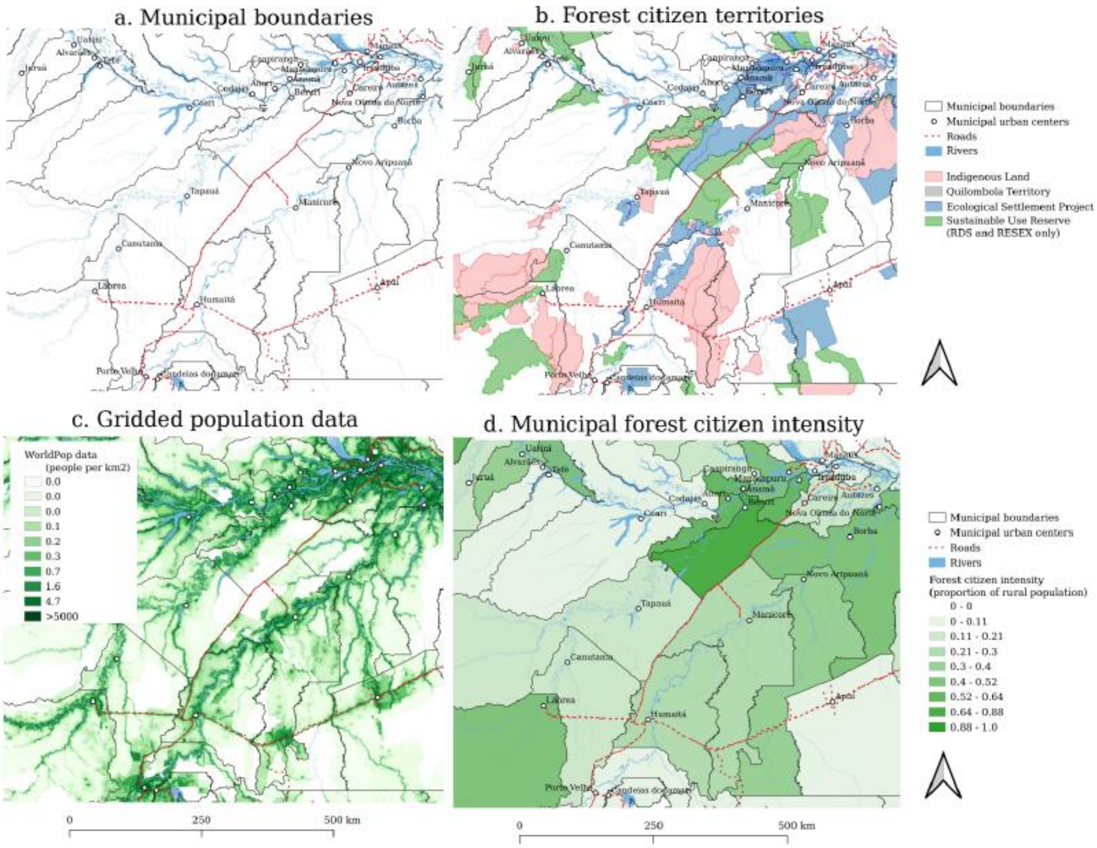

Appendix S4. Maps illustrating our method for calculating the forest citizen population within selected territorial categories using WorldPop gridded population data, and then calculating municipality-scale forest citizen intensity. The maps show part of Amazonas state, with Rondonia state below.

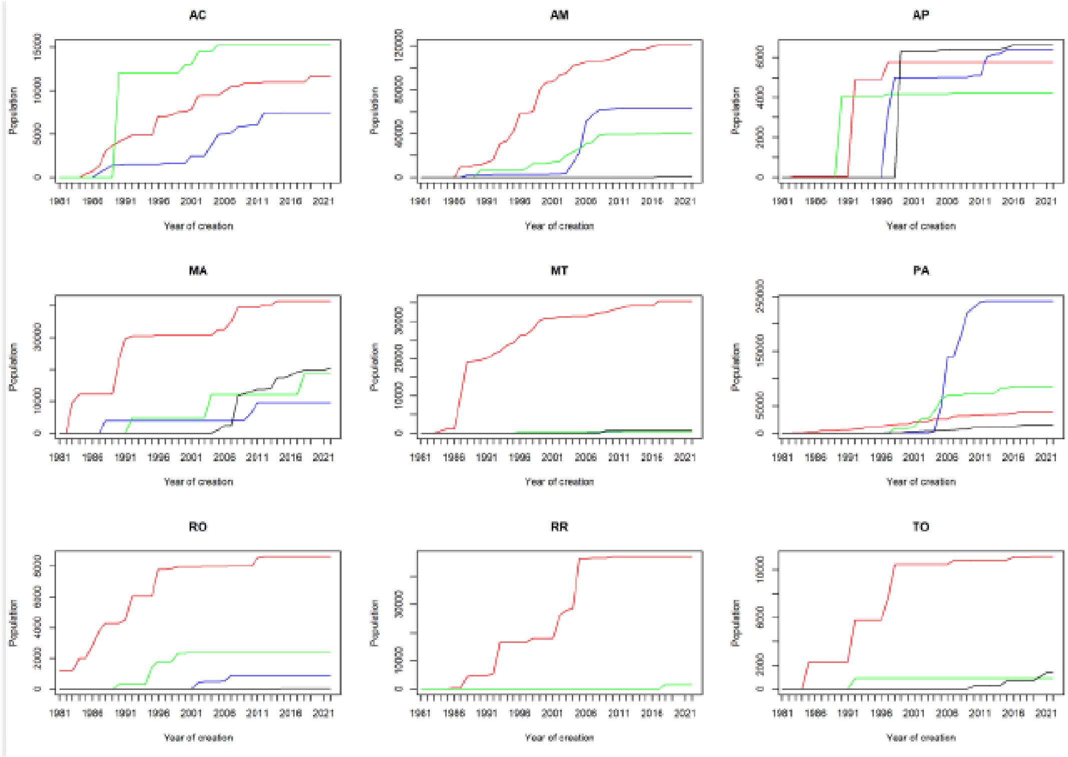

Appendix S5. Change (1981-2021) in the total resident population in forest citizen territories, separated by State. Populations estimated by intersecting the boundaries of territories created in a given year (for Indigenous Lands (ILs) and QTs; from phase onwards) with WorldPop gridded population data (from 2010). Red lines refer to ILs, green to RDS and RESEX reserves, blue to Ecological Settlement Projects, and black to QTs. Letters refer to states (Acre (AC), Amazonas (AM), Amapá (AP), Mato Grosso (MT), Pará (PA), Roraima (RR), Rondônia (RO), Tocantins (TO).

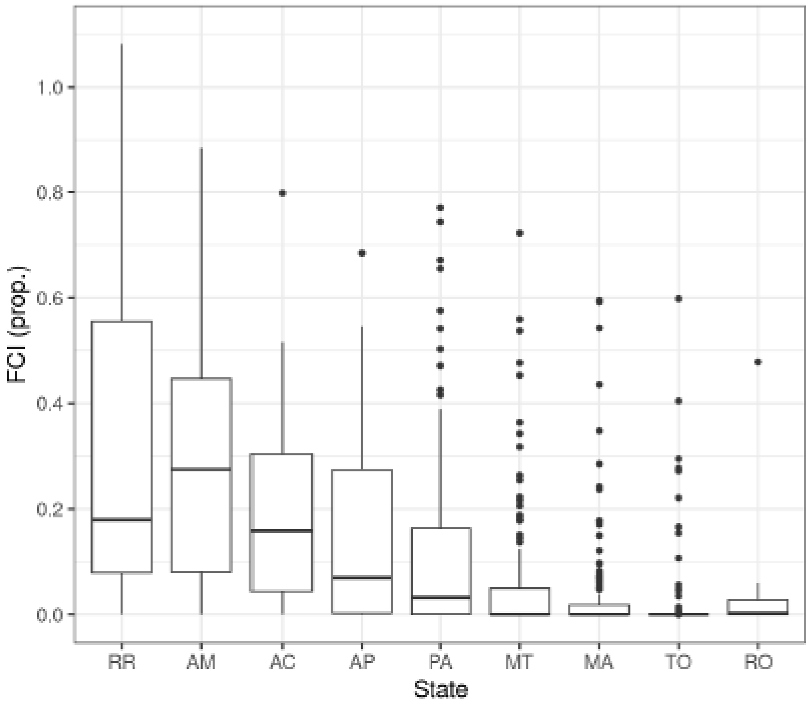

Appendix S6. Boxplot showing variance in municipality-scale forest citizen intensity (FCI) across states in the Brazilian Amazon in 2020. FCI is calculated as the proportion of the rural population in each municipality (estimated using WorldPop data) residing in the territorial categories defined as fostering forest citizenship. Letters refer to states (Acre (AC), Amazonas (AM), Amapá (AP), Mato Grosso (MT), Pará (PA), Roraima (RR), Rondônia (RO), Tocantins (TO).

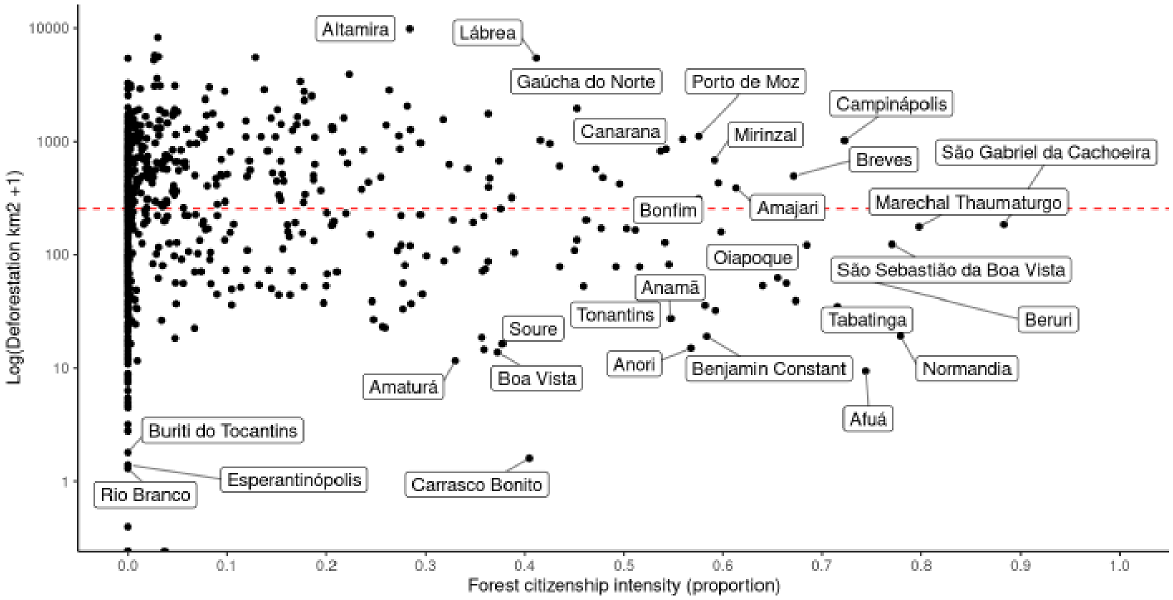

Appendix S7. Scatterplot showing forest citizen intensity (FCI) in relation to 21st Century areal extent of deforestation in municipalities in the Legal Amazon. Despite the very high variance, there is a significant positive correlation between FCI and deforestation amount (0.1231734; $p < 0.001$, Kendall correlation). The dashed red line shows median deforestation (257 km2). Labels refer to the names of municipalities.

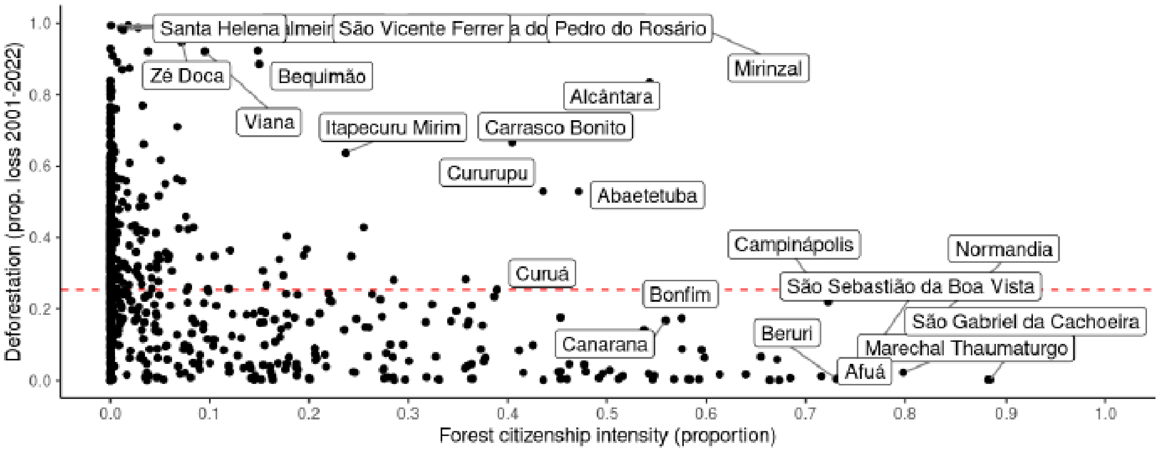

Appendix S8. Scatterplot showing forest citizen intensity (FCI) in relation to 21st Century deforestation in municipalities in the Legal Amazon, as a proportion of forest remaining in 2001. Two outliers with very high deforestation were removed. There is a significant negative correlation between FCI and deforestation proportion (-0.3295384; p <0.001, Kendall correlation). The dashed red line shows the median proportion (0.255) of remaining forest lost from 2001 to 2022 .

Appendix S9. Territorial analysis (creation, implementation, on-going challenges to claiming rights)

9.1. Territorial analysis methods

Specific encounters discussing forest citizenship in theory and practice included multiinstitutional workshops in Brazil in 08/2022 and 10/2022, interactions with postgraduate students in Amazonian Agricultures at the Federal University of Pará (09/2023), a workshop with researchers and practitioners at the Mamirauá Institute for Sustainable Development, Tefé (11/2023), and multiple online and in-person conversations between the research team since 07/2021. Whilst the purpose of this paper is not to present detailed results and our conceptualization of forest citizenship has been informed by decades of fieldwork across Amazonia, it has benefited from qualitative fieldwork specific to this topic between 05/2023 and 02/2024 in the RESEX Rio Iriri in Pará State, urban fieldwork in Altamira in Pará, in communities on the Manicoré River in Amazonas, in RDS Amanã in Amazonas, and in RDS Mamirauá. This included diverse interactions with rural communities, Amazonian NGOs, public employees, etc. L.P. also draws on experiences with the Amazonas Citizens Network which he coestablished (with N. Filizola, UFAM) for collaborative workshops between 2015 and 2017, drawing together grassroots perspectives from four municipalities in Amazonas; Caapiranga, Jutai, Ipixuna, and Maués. The encounters 2022-present fit under the umbrella of a project under the Trans-Atlantic Platform for Social Sciences and Humanities, titled: Forest Citizenship for Disaster Resilience: learning from COVID-19 (FORTE). This project involves UK, US and Brazilian institutions, the latter in Sao Paulo State, and two Amazonian States of Pará and Amazonas. This project has been approved by the Lancaster University Research Ethics Committee (reference FST-2023-3453-RECR-4) and adheres to the Ethical Code of the Brazilian Sociological Society. Permissions to enter forest territories included SISBIO 87493-1, and SEMA SIGED No 01.01.030101.006835/2023-53.

9.2. Creation processes of the included territorial categories

(a) Formal steps in the creation and implementation process for federal Extractivist Reserves (RESEX):

(1) Written demands by traditional communities;

(2) Creation of residents association, which then needs to formally decide to pursue this kind of territory;

(3) State-sponsored expeditions to survey biodiversity, habitats and community socio-economic/demographic characteristics;

(4) public consultation (*consulta publica*);

(5) creation decree (*decreto de criação*);

followed by these implementation phases:

(6) creation of a Deliberative Council (*Conselho Deliberativo*);

(7) development of a Management Agreement (*Acordo de Gestão*);

(8) completion of a participatory Management Plan (*Plano de Manejo*) which is the main management tool for this kind of protected area

(9) approval of the Management Plan by the Deliberative Council.

The participatory management plan should be updated every three years and include a shared mission and future vision statement, and define programs around social and environmental sustainability co-constructed with the traditional population (Instrução Normativa ICMBio N° 01/2007). These reserves have buffer zones. Communities specify natural resource management rules around hunting, fishing for food, and ornamental fishing. Reserves have rules on forest clearance per family, restrictions on raising-cattle, and rules on new arrivals. Members can create Work Groups around gender, age, or livelihoods. For instance, artisans may work collaboratively, including strategies for accessing markets and negotiating prices and commercial relationships.

Government agencies are responsible for the expropriation of private, titled land overlapping with RESEX boundaries.

(b) Formal steps in the creation and implementation process for state Extractive Reserves (RESEX) in Amazonas.

(1) Joint request for a RESEX by riverine communities and state institutions

(2) Approval of the proposal by a group representing the resident population

(3) Social and biodiversity surveys

(4) Public consultation (*Consulta publica*) or Public hearing (*Audencia publica*)
(5) Signed community request for the reserve
(6) State decree to create the RESEX.
Followed by these implementation phases
(7) Creation of a residents association (*Associação de Moradores*), followed by leadership elections for the Directorship and sector-specific leaders.
(8) Creation of deliberative management council
(9) Participatory development and approval of a Management Plan (*Plano de Gestão*)

Creation of Sustainable Development Reserves (RDS) in Amazonas follow broadly similar steps as specified in Amazonas State Lei Complementary No. 53, 5 June 2007.

(c) Formal steps in the creation and implementation process for Indigenous Lands (*Terras Indigenas*):
(1) Self-recognition as indigenous by a community or communities
(2) Declared (i.e, *declarada*) through approval by the Ministry of Justice.
(3) Under study (*em estudo*). By a FUNAI Work Group (Grupo de Trabalho) which submits a report including the resident indigenous group(s), and the proposed area and spatial boundaries. Under Brazilian law and when applied to territorial disputes around mega-projects, etc, territories in this phase are not yet considered Indigenous Lands because the State has not yet recognized particular spatial boundaries. Nor does the state engage in any enforcement actions to protect territorial integrity, at this stage.
(4) Delimited (i.e., *delimitada*) spatial boundaries. With the Work Group's contributions complete and approved by the Presidency of FUNAI, a key report is published (RCID; *Relatório Circunstanciado de Identificação e Delimitação*). At this point, the territorial limits have been officially recognized and receive judicial guarantees from the Ministry of Justice.
(5) "Forwarded" as RI (encaminhada RI)
(6) Homologated (homologada) by Presidential decree
(7) Regularized (*regularizada*)

(d) Formal steps in the creation and implementation process for Quilombola Territories: INCRA Normative Instruction No. 57, of 2009 - establishes procedures for recognizing Quilombola Territories.

1) Certification. The community's self-definition as a remnant of quilombo communities must be certified by the Palmares Cultural Foundation, by means of a Certificate of Registration in the Foundation's General Register of Remnants of Quilombo Communities.
2) Opening of the administrative process with INCRA by means of a request from any interested party or representative association.
3) Identification and delimitation - studies that will analyze the land claimed, drawn up by an interdisciplinary Technical Group appointed by Incra's Regional Superintendence. The identification of the boundaries of the lands of the remaining quilombo communities must be based on indications from the community itself, as well as on technical and scientific studies, including anthropological reports, and will consist of the spatial, economic, environmental and socio-cultural characterization of the land occupied by the community, consolidated by means of the Technical Report on Identification and Delimitation (RTID).
4) Publicity - the RTID will be submitted for preliminary analysis by INCRA's Regional Decision Committee which, once it has verified that the criteria established for its preparation have been met, will send it to the Regional Superintendent for preparation and publication of the public notice, twice consecutively, in the Federal Official Gazette and in the Official Gazette of the federal unit where the area under study is located.
5) Consultation with bodies and entities - At the same time as its publication, the RTID will be sent to a series of bodies and entities so that, within a common period of 30 (thirty) days, they can present their opinions on the matters within their respective competencies.
6) Challenges - Interested parties will have a period of ninety days after publication and notification to challenge the RTID at Incra's Regional Superintendence, attaching the relevant evidence.

7) Analysis of the land situation of the areas claimed
8) Demarcation - physical demarcation of the area.
9) Titling - The president of INCRA will carry out the titling by granting a collective and pro-individual title to the community, in the name of their legally constituted association, without any financial burden, with the obligatory insertion of an inalienability, imprescriptibility and unseizability clause, duly registered with the Registry Service of the District where the areas are located.

e) Formal steps in the creation and implementation of Ecological Settlement Projects:

Incra Normative Instruction No. 129, of 2022 - establishes administrative procedures for the creation by Incra of settlement projects and environmentally differentiated settlement projects.
(1) Formalization by INCRA, in the Electronic Information System - SEI, of a specific administrative process for the creation of a settlement project or environmentally differentiated settlement project, accompanied by the following documents.
(2) Preparation of studies, with a conclusive opinion on various aspects of the property and the settlement project, such as the project area, the Income Generation Capacity Study (ECGR), how the property was obtained, etc.
(3) Preparation of a draft decree creating the settlement project.
(4) Send notice to the municipality where the settlement is located, informing them of the proposal to create a settlement project in the region.
(5) Submission of the proposal to create the settlement project to the Directorate for the Development and Consolidation of Settlement Projects - DD, by Decision Order of the Regional Superintendent.
(6) Analysis, by the Directorate for the Development and Consolidation of Settlement Projects - DD, of the conformity of the proposal to create the settlement project and authorization of the creation of the settlement project by Decision Order of the director, submitting it for approval by

the President of INCRA.
(7) The president of INCRA approves the creation of the settlement project by issuing an Ordinance creating the settlement project.
(8) Publication of the Ordinance in the Federal Official Gazette.

9.3 Time from proposal to creation of territories.

For example, the RESEX Unini, a sub-tributary of the Rio Negro in Amazonas State. Following creation of a Residents Association and proposal for creating a projected area in 2002, the RESEX was created by Decree in 06/2006, its Deliberative (management) Council – essential for implementation – was created in 11/2009, and its first Management Plan was approved in 10/2014.

9.4. Role of external institutions and individuals in the community mobilization process.

Various kinds of institutions, such as churches, universities, unions and nongovernmental organizations can act as co-protagonists in the process of creating a particular forest territory. During the initial stages, different institutions, whether overtly political or not, and in more or less active ways, may cooperate with the communities fighting for territorial recognition in order to consolidate a political organization. It is important to note, however, it would be impossible for any external institution to achieve sufficient local mobilization, commonly involving risks and commitments, including confrontation with powerful local oligarchies, alone. Rather, it is invariably leaders and activists from the forest peoples themselves that spearhead the struggle for recognition in the forest, and all too often suffer violence as a consequence (Butt et al 2019). Hence decision-making should remain with communities themselves and their associations. Indeed, some ‘external’ institutions such as churches are also part of communities in the sense there may be small churches or chapels, and community residents can have roles as an evangelical pastor or leader (*dirigente*) or catholic prayer-leader (*catacista*). Those opposing the recognition of forest-dwellers’ rights often claim that Quilombolas, indigenous people, or *ribeirinhos* are merely puppets under the sway of external agents, and hence advancing the agendas of environmentalists, left-wing political parties, churches, unions, universities, etc. We

reject this as prejudiced reductionism, since this leads us to understand such groups of forest-dwellers as incapable of making choices, of being merely the subjects of social and social processes and transformations, as if the capacity for thought and action cannot exist outside elite social spheres.

The creation process of the RESEX Unini, Amazonia, exemplifies how NGOs may partner with communities in their attempts to collectively organize to achieve territorial recognition. The socio-environmental NGO *Fundação Vitória Amazônica* (FVA) supported communities on the Rio Unini to develop a residents association (Associação de Moradores do Rio Unini - AMORU) in 2002 to tackle predatory fishing and government attempts to limit access to the river (Plano de Manejo 2014). Communities worked collectively to seek a sustainable use territory; they decided on a RESEX and succeeded with creation of the RESEX Unini in 2006.

An evangelical pastor, Antônio Vasconcelos, was instrumental in working alongside riverine community to create a religious-based Agroextractivists Association in 1997 (APADRIT - Associação dos Produtores Agroextrativistas da Assembleia de Deus do Rio Ituxi) in Lábrea, Amazonas, and by 2008, the creation of the RESEX Ituxi. ADAPRIT worked closely with partners in other institutions including the *Conselho Nacional das Populações Tradicionais* (CNS, formerly the rubber-tappers union), the federal environment agency IBAMA, a pan-Amazonian association, the *Grupo de Trabalho Amazônico* (GTA), and a land-rights institution within the Catholic church, *Comissão Pastoral da Terra* (CPT)(Aleixo & APADRIT, 2011). The protagonists later framed achieving territorial creation process in terms a longer-term process of actualizing citizenship:

"The official creation of the Ituxi River Extractive Reserve was received as a victory. However, this was quickly perceived as just one important step in an ***even longer and more difficult process****: the* ***affirmation of citizenship and dignity*** *of families on the Ituxi River, based on the conservation of the region's forest and natural resources."* Aleixo & ADADRIT, 2011. p9.
(our translation from Portuguese, and added emphasis)

For instance, from the 1960s onwards the catholic church in Tefé, Amazonas, played a role as co-protagonist in the emergence of community mobilization and organization in this region. Rubber-tapping households began to relocate from *sitios* (isolated settlements often deep in the forest) and form communities at lake-sides or edges of rivers, and work cooperatively to achieve common goals. These community-based movements later contributed to the creation of the RDS Amanã (Plano de Gestão 2019). For some QT and IL, anthropologists and other social researchers have been co-protagonists in the process of self-recognition and formal declaration of land-claims. In some instances, the state has taken the lead role in the creation of forest territories, approaching communities to garner interest in a RDS, for example. There are several examples of this in Amazonas, where a period of ambitious state-appetite for creating sustainable use reserves was perhaps faster (in some cases) than bottom-up demand.

For instance, the state RESEX Rio Gregorio, Amazonas was initially proposed in response to governmental concern (voiced at seminar Macapá in 2002) about advancing frontiers of illegal logging and cattle-ranching in that area. Then, somewhat unusually; "*in 2004, two grandchildren of the former owner of the Santo Amaro rubber plantation ... born on the Gregório River and working in State institutions, took the initiative to create an Extractivist Reserve on that river*" (Plano de Gestão 2010). Only later, in 2005, was a proposal developed with the state government approved by the Residents Association of the AgroExtractivist Settlement Project Santo Antônio–Mourão (AMPAESAM), a *PAE* kind of ecological settlement in Eirunepe, Amazonas. Other examples with questionable levels of bottom-up mobilization include several territories around the BR-319 mosaic in southern Amazonas – with tangible concerns about advancing deforestation frontiers. This includes territories created by Amazonas state in the municipalities of Apuí e Novo Aripuanã which had no residents, only users (e.g. RESEX Guariba, RDS Aripuanã, and RDS Bararati).

9.5. Multiple territories from one association

A residents or producers association may eventually succeed in creating several territories. For instance, the Association of Rural Producers of Carauari (ASPROC, Rio Juruá, Amazonas) was created in 1994, describes itself as "*an organized struggle by agroextractivist producers for survival in the forest and environmental conservation"*

(https://asproc.org.br/a-asproc/#), whose central aim is “*promoting citizenship for indigenous peoples and traditional communities in the mid (River) Juruá*”. ASPROC was heavily involved in the creation of RDS Uacari and RESEX Médio Juruá. In the words of ASPROC elected President, Manuel Siqueira (Manuelzinho) in 2020:

“*Things happened in stages, first was this process of us* ***organizing ourselves into communities to seek our rights****, the second step was* ***creating an association that represented us*** *and the other step was the* ***fight for territory****, because we could only move forward if we were able to guarantee the territory. When it was created, ASPROC also had the objective of fighting to guarantee the territory. So ASPROC was super important in this process, I would say that there would not have been the creation of RESEX if we hadn't created ASPROC before. RESEX was created in 1997 and ASPROC continued to work in the process of organization and social mobilization, providing full support so that RDS Uacari could also be implemented, covering communities that had been left out of RESEX, and together we guaranteed 100% of the territory for communities. So, ASPROC was super important to guarantee this mobilization, this search, this articulation and interconnection of conflicts so that we could reach our objective, which was the implementation of RDS Uacari.*“ (Interview with C. Machado, 2020, our translation and added emphasis)

9.6. Threats motivating community mobilization

Wild-cat gold-mining was a major community concern motivating the creation of numerous reserves (e.g., RDS Aripuanã, RESEX Guariba), ecological settlements (e.g., PAE Lago Grande), and IL (e.g., TI Coatá-Laranjal). Communities also mobilized in response to fishing conflicts, particularly external, urban-based commercial fishers (e.g, RDS Rio Madeira; and for RESEX Unini, sports-fishing by tourists) depleting fish stocks in their lakes and rivers. Irregular, unauthorized hunting of terrestrial animals by outsiders was also mentioned during the creation of RESEX Unini and RESEX Rio Gregório, and TI Vale do Javari. In specific cases, communities felt their livelihoods and cultural practices were threatened by outsiders harvesting river-turtles (e.g., RESEX Catuá Ipixuna), and non-timber forest products such as Brazil nuts (e.g., TI

Caititu). A more widespread threat was from illegal logging of valuable hardwoods. In some cases, illegal logging was selective (leaving the forest structure broadly intact) (e.g., RESEX Rio Gregório, or TI Vale do Javari), whereas many other communities/areas were threatened by logging in conjunction with land-grabs (*grilagem*), and clear-cut deforestation for creating cattle-pasture (RESEX Catuá Ipixuna, TI Jauary) and land speculation, and related conflicts. Although there is a weak positive correlation between cumulative deforestation since 2001 and municipality-scale FCI, the location of forest citizenship territories appears to vary greatly in relation to active deforestation frontiers (Appendix S5,S6, main paper Figure 1). Concerns over the social and environmental impacts of larger-scale governmental projects, including highway development (e.g. paving BR-319 in Amazonas [RESEX Canutama] or the new bridge across the Rio Negro [RDS Rio Negro]), was central to the creation of several territories. Community resistance to government-sanctioned plans for large-scale commercial mining of iron-ore and potassium contributed to the creation of QT Saracura, and TI Jauary. Another example of resistance to the state is community mobilization on the Rio Unini, for a RESEX, against environmental agency IBAMA attempts to block access to the river with a metal chain. Stated justification for some territories was to confront the invisibility of their rural populations to the Brazilian state, which manifests in deficiencies in public service provision, technical support for livelihoods, access to welfare programs, or precarious transport connections (e.g. unpaved roads) (e.g., PAE Maripiti).

9.7. On-going rights struggles

9.7.1. Maintaining social and political organization.

All the selected territories are managed through *associativismo;* the movement or practice of uniting to work collectively to defend the interests of all residents, or particular groups such as a livelihood category, or mothers, etc. Within territories, communities normally hold elections in order to select their leadership including a President, Vice-President, Secretary, Treasurer, and a Fiscal Council whose main task is overseeing financial matters. In indigenous communities, instead of a President there is normally a Tuxaua or Cacique. Not-for-profit associations such as a community's

Resident Association are required to officially register, pay an annual tax (or incur fines), and (in effect) contract an urban bureaucrat to declare any changes in committee composition following elections. Some communities have formal statues, laying out objectives, rules and members' duties. Community leaders are responsible for internal issues (e.g., organizing financial contributions to run a community electricity generator in the evenings, or resolving land disputes around farm-fallow agriculture and family use-rights to particular areas), resolving any conflicts with neighboring communities, and also to represent community interests in sector-specific, wholeterritory meetings, or further afield. Mathews (2021) frames these leadership challenges in rural Amazonia in terms of the necessity of creating and maintaining social capital configurations, or seeking and attracting social relationships. Specifically, bonding ties within communities, bridging ties with neighboring communities, and linking ties in local urban centers and larger cities.

Territories may have specific work groups responsible for particular livelihoods (e.g. handicraft artisans) or running a Brazil nut processing factory, for example. Associations normally raise their own funds, liaise with other organizations (e.g., nongovernmental organizations) and may have to navigate other bureaucratic processes in order to access credit, or receive external recognition. Many territories have higherlevel Residents Associations with representation from a range of communities. These representatives are responsible for implementing management plans, in conjunction with government institutions, whilst making sure that communities' voices are heard within the decision-making structures of territories (e.g. each RESEX has an annual General Assembly).

Community leaders are also closely involved in bottom-up processes of selfrecognition as indigenous people or Quilombolas, which may involve navigating through the process of a community wishing to be removed from a particular territorial category and instead receive alternative territorial recognition. For example, 11 communities in the RDS Amanã formally self-declared as indigenous between 1994 and 2019 (Plano de Gestão 2019). The need to cooperate with other communities and institutions to collectively manage fisheries and other natural resources continues even after the completion of leaving an RDS and becoming an IL (Zanatto & Rosa 2023).

9.7.2. Access to basic public services, principally education and healthcare.

Brazil is highly decentralized and municipal governments (*prefeituras*) are responsible for delivering most public services. The mayor (*prefeito/a*) holds a lot of power and communities must negotiate service access either directly with the mayor, or with the person in charge of a particular Secretariat (e.g. the health secretary), or with one of their team (e.g. a sector's education coordinator). Most of these negotiations are done in towns, at the convenience of those in charge. Albeit some community leaders in the RDS Mamirauá, for example, directly communicate with the mayor by Whatsapp, and Starlink internet.

Unmet demand for education is an on-going driver of rural-urban migration in Amazonia, including from within recognized territories (e.g., RDS Amanã, Amazonas - Pereira et al. 2022). Dodd (2020) found that a desire to access highschool education explained, in part, why many households in the RESEX Gurupá-Melgaço (Pará state) were multi-local, with some household members mainly living in the nearest town, and others in the Reserve. Rural Amazonian communities must negotiate with their municipal government for elementary education access, which at a basic level is about acquiring or continuing to have a nearby school. Insufficient numbers of school-age kids, or political conflicts, may prompt the local education secretariat to propose schoolclosure. Additional struggles for elementary education center on the quality, size and adequacy of the school itself, or lobbying to achieve power (through a generator), computing, printers, internet, a kitchen, toilet, clean water, sufficient quantity and quality of school meals, staffing of teachers and support staff, night-school for youths and adults, qualified teachers, tackling absenteeism from teachers sent from the local town. School quality may be limited by broader deficiencies in community infrastructure, unreliable electricity supply or lack of piped, clean water. High-schools are relatively uncommon in rural Amazonia, and may be taught remotely by satellite, on a television screen, with an *in situ* facilitator (Nascimento 2017). High-school education is under the remit of state governments, managed through an office in the local town.

Where a community lacks its own elementary or high-school, the community may need

to negotiate fluvial transport (ranging from fuel contributions only, to salaried boatdrivers and boats) with municipal and state education authorities. It is not uncommon for public employees in some municipalities going months without pay, and getting paid can be a major source of stress and necessity to travel from communities to town (beyond the normal weekly or monthly visit).

Rural Amazonian communities have historically received limited i*n situ* healthcare and river-dwellers have instead had to make (often difficult, time-consuming and expensive) journals to urban centers. Access to healthcare in Amazonia - in both rural and urban locations, is intertwined with political power (Abel 2022) and rural healthcare remains precarious (Garnelo et al. 2020). Luna (2009) analyzes the normalization of social and health inequities in Brazil, including ways in which marginalized Brazilians are degraded, stigmatized, humiliated and 'let die' – in a Foucauldian sense – by the national universal health system, the SUS.

Communities may need to pressure municipal health authorities to obtain a community health agent (normally a local community member, often working in several small communities), or other resources including a healthpost with nursing technician or nurse, microscope operator (particularly for diagnosing malaria) and related consumables, and '*ambulancha*' speedboats for urgent transport of patients to the nearest urban center (Almeida et al. 2022). Communities may need to negotiate visitation by boat-based well-equipped health teams with doctors, dentists, antenatal care professionals, and others (UBS Fluvial), and vaccine teams for infectious diseases (el Kadri et al. 2019). At least outside of legally-recognized territories, more remote rural communities in sparsely-populated areas have been much less likely to have functioning schools, health-agents, electricity, clean water (Parry et al. 2010). Communities in the process of being recognized as indigenous must obtain official permission from the *Registro Administrativo de Nascimento Indígena* [RANI] in order to obtain healthcare reserved for indigenous peoples, from the *Distrito Sanitário Especial Indígena* (DSEI). This approval is not always straight-forward (e.g., https://terrasindigenas.org.br/pt-br/noticia/178444). Preventative public health measures also require significant energies from community members and leaders in order to obtain investments such as wells (reducing dependence on river-water, and

related health risks and labor spent on collecting), water filters, pumps and water storage (requiring electricity), toilets in shared spaces such as schools.

Accessing public services and the welfare state (see below), along with trading and shopping opportunities, strongly motivate rural-urban mobilities in Amazonia. An individual or household may travel alone (e.g. in a motorized canoe) or pay for a ride in a larger boat belonging to a community member or trader. Whichever the case, more remote households face longer, more expensive journeys, and therefore tend to travel to urban centers less often than households closer to town (Parry et al. 2010). Riverine populations face greater difficulties (risks of accidents, fuel consumption, time) in reaching towns during the dry season (Parry et al. 2010), whereas communities connected by unpaved tracks and roads (e.g., RESEX Chico Mendes in Acre) face greater challenges during the wet (rainy) season.

9.7.3. *Access to government welfare programs*.

Rural access to supposedly universal welfare programs has historically been challenging due to the interplay of extremely limited presence of the state in rural areas, and concomitant lack of rural citizens' visibility to the state due to lack of documents such as birth certificate (*registro de nascimento*), taxpayer identification card [*Cadastro de Pessoas Físicas*, or 'CPF'], bank account and card, electoral registration (*Titulo de Eleitor*); official employment record (*Carteira de Trabalho*); identity card (*Registro Geral* or, 'RG']); health system card (*Carteira Nacional de Saúd*e), boat pilot's license, etc. Beyond documents, access to programs and welfare may be hampered by illiteracy and/or stigmatization in public offices. Hence the social inclusion offered by territorial recognition may include help with registration processes (in retirement pensions linked to being a rural producer; Forest Grant; incapacity benefit; maternity salary, etc) and perhaps community transport to receive payments in the nearest town. For instance, Parry et al (2010) found that, despite high rates of poverty in remote locations beyond 100 km from Amazonian urban centers, in 2007 only 11% of families in these places were receiving the conditional cash transfer Bolsa Familia, compared to 49% of families <100km from towns. Hence an important task, and goal, of territorial recognition is increasing access to government programs. This is

demonstrated clearly by the affirmation of the creation of PAE Maripiti, justified by the head of INCRA Amazonas in terms of citizenship:

"*With the creation of the settlement, **these people will be recognized as citizens**. They will be able to obtain their documents, have access to support credits and improve, mainly, their housing, which is very precarious*."
J. C. Serra Gonçalves, 2007. (our emphasis added)
(https://www.agrisustentavel.com/floresta/agroextra.html).

9.7.4. Fair access to markets, improved incomes and livelihoods.

This confronts the long-term exploitation of traditional Amazonians within credit-based trading systems centered on natural resources. For example, in the *aviamento* rubber economy, rubber-tappers were often coerced to live in very isolated locations and trapped in debt-peonage; forced to exchange low-returns on their labor for purchasing food and other essentials (Mathews & Schmink 2015). Other examples include receiving very low prices for Brazil nuts, or buying staples and selling diverse forms of produce to riverine *regatão* traders. Finally, commercial fish-buyers such as on the Rio Solimões are infamous for their cartel-like behavior in price-fixing to lower the prices they pay to fishers (L.P., personal observation). Outside of territories, trading conditions get more unfavorable with greater distance from urban centers (people paid less for their agricultural and forest products, and charged more in return for staple goods; Parry et al. 2010). In some territories, associations attempt to reduce spatial variation in these trading conditions to ensure fair prices for harvested natural resources (e.g., fish, tree oils) and provide rural access to affordable foodstuffs and other staples. For example, ASPROC on the Rio Juruá, Amazonas, has a large-scale operation with its own fleet of boats operating for thousands of kilometers from Manaus, rural trading posts with fixed prices and focusing on ensuring equity across the value chains for *Arapaima* fish, and tapped-rubber including collective bargaining with buyers.

Territories can allow for institutional innovations in environmental legislation, allowing multiple communities to partner with governmental and non-governmental institutions in order to gain permission for sustainably harvesting wild species whose

trade is otherwise illegal. For example, caiman management in the RDS Mamirauá, community management of pirarucu fish (*Arapaima gigas*) in lakes (Campos-Silva & Peres 2016), and river turtles. Territories also provide a platform for seeking collective large-scale investments in livelihoods, such as in a Brazil nut processing factory (attempting to sell high-graded, dried nuts for export; see Le Tourneau & Greissing [2010] for an insightful case study from the RDS Iratapuru, Amapá State), or access to other kinds of machinery (whether agricultural or for processing wild-harvested plant or animal products) or credit. Often, livelihood investments are supported by regional, Brazilian, and/or international NGOs. The Amazonas State government and Fundação Amazônia Sustentável maintained the *Bolsa Floresta* program between 2008 and 2022, providing residents of many state-created RDS and RESEX territories with a monthly cash payment (R$50). And partnering with Residents Associations and Producers Associations to invest in livelihoods (Viana et al. 2021). The latter includes commodity chains related to community-based tourism, handicrafts, *açaí*, cocoa, *farinha* (toasted manioc flour), bee-keeping and honey, amongst others. *Bolsa Floresta* is being replaced by *Guardiões da Floresta*, whose implementation had not started, as of 02/2024. There are pockets of strong ecotourism in some parts of the Brazilian Amazon (e.g. many coastal areas in Pará State; on Ilha do Marajó; around Santarém; around Manaus, around Tefé) and community-level and territory-level organization can be important in helping communities to develop their own benefits (e.g. through acquiring capital for a community lodge [pousada], a handicrafts center, or negotiating a fair share of revenue from high-end sports fishing by outsiders).

9.7.5. Conserving forests and biodiversity through sustainable resource use.

The impotence of individual forest communities to resist illegal logging and land-grabs, frequently financed by powerful regional elites, motivates many bottom-up demands for territorial recognition (e.g. RESEX Ituxi, Amazonas). In many cases, environmental threats linked to resource grabs interlink with government actions or inaction to pose tangible risk to forest-dwellers' lives. For instance, the deaths of Yanomami indigenous people during Bolsonaro's Presidency, due to the intersection of violent intent from gold-miners and systematic state neglect of their harm and suffering (Lobo & Cardoso 2023). Territorial recognition normally places considerable additional individual and

collective environmental responsibilities on rural communities. In some territorial categories, the Management Plans – which must be developed with, and approved by, residents – specify limits per household on annual forest clearance for planting manioc and other crops. And there may be total or partial restrictions on raising cattle, and hence, avoiding deforestation to create new pastures.

Communities must also work together to manage wildlife harvest (in terms of spatial, temporal and species controls) and any restrictions on commercialization/sale. Enforcing fishing agreements – such as managing ox-bow lakes to manage *Arapaima* populations – can be rewarding eventually but often demands very large time commitments (e.g. in the RDS Mamirauá, people often take it in turns to spend a week or more at a time away from home) to monitor the lakes and deny access to opportunistic outsiders. Specific shared environmental responsibilities in territories can include conserving threatened species (e.g. river turtles; limiting consumption, releasing juveniles, protecting and monitoring nesting beaches). Additional responsibilities can include keeping out loggers, and gold-miners. Examples of external efforts to foster environmental leadership with forest territories include the federallyfunded program of Young Communicators (formerly, Young Protagonists) in RESEXs (e.g. in the RESEX Unini, Amazonas), sometimes delivered in conjunction with NGOs. These investments in pro-environmental values, attitudes and behaviors are akin to fostering ecological citizenship in the sense of duties and responsibilities.

9.7.6. Coping with disasters.

Drought conditions restrict riverine navigation and hence, rural-urban mobility (e.g., many communities in the RDS Amanã, Amazonas, were effectively cut-off from the urban center of Tefé for several months during the major drought in 2023 (L.P., personal observation). Changing hydrological conditions are causing high rates of riverbank collapse and loss of buildings in Amazonas State (A. Fleischmann, personal communication). Some riverine communities are adapting by deconstructing buildings and relocating (our observations from RDS Mamirauá). Conversely, high rates of sedimentation are causing other rural communities in this RDS to become farther from the river edge, posing a major time burden on accessing boats and canoes for

livelihoods and rural-urban mobility (A. Fleischmann, personal communication). Changes to hydrological seasonality also imposes impacts on farming and fish management in lakes (A. Fleischmann, personal communication). Additional risks include outbreaks of climate-sensitive diseases (e.g. through effects on insect vectors), scarcity of clean water during extreme dry seasons, crop failure through drought or flooding, or the COVID-19 pandemic. High social vulnerability of Amazonians amplifies risks of harm from climatic extremes (Parry et al. 2018) including the effects of periods of extremely low or intense extreme rainfall on human health (Chacon-Montalvan et al 2021). Parry et al (2019) argue that the climate-health risks facing rural Amazonians are neglected and under-estimated due to interacting forms of invisibility, including social and spatial marginalization within Brazilian society and health systems, neglected diseases, and under-resourcing of mental health.

Rural forest-dwellers in the Brazilian Amazon are increasingly affected, directly and indirectly, by a violence epidemic associated with drug-trafficking, sometimes intermixed with piracy. For instance, in Amazonas state, people living between the trifrontier Brazil-Colombia-Peruvian border, and down the Rio Solimões – including residents of territories such as the RESEX Auatí-Paraná, IL Cuiú-Cuiú, and RDS Mamirauá, are caught up in the violent encounters between Colombian drug-traffickers (often coming down the Rio Japurá sub-tributary) and Brazilian criminal factions and militias, gold-miners and river pirates (Perez & Filho 2024). Homicide rates increased markedly for most provincial municipalities (i.e., excluding the capital Manaus) in Amazonas State between 2009 and 2020 (IPEA 2023). See also Gonçalves and Ferreira (2020). Nonetheless, communities are responding, including cases of members becoming trained, registered Municipal Guards (*Guarda Municipal*) sometimes with the right to bear a fire-farm.

Friction around identity based rights

Forest-dwellers are fighting for multiple rights simultaneously, both pre, during and post-creation of a recognized territory. For residents of the RESEX Médio Purus, officially created in 2008, in Lábrea, Amazonas, the principal motivations for riverdwellers seeking territorial recognition were (i) overcoming their invisibility to the state

(and receiving public services), (ii) ending economic exploitation by traders in nontimber forest forests, including memories of violent oppression by rubber bosses (*patrões*), and (iii) being empowered to (favorably) end conflicts with commercial fishers and loggers (Aleixo and ATAMP, 2011). Bottom-up resistance from dozens of communities, with the support of the catholic land-rights institution CPT and the nongovernmental Amazonian Work Group (GTA), coalesced in the creation of their 'mother association' (*associação-mãe*) in 2004; the Association of the Agroextractivist Works of the Médio Purus (ATAMP). Nonetheless, motivations for some involved included the perception that other groups of forest-dwellers have greater rights (implicit is the right to land, natural resources and - in our experiences, a perception of non-tribal riverdwellers that indigenous people have much greater access to public services), at their expense:

"With the RESEX, things are, let's say, 25% better. Before the struggle [luta], *we didn't know a third of what we know today. We did not have dialogue to talk to anyone, **we didn't know our rights**. The **Indian mocked me*** [babava na minha cara] *and I didn't say anything because I didn't know my right, the right I had there in my community. So-and-so doesn't turn up there today saying: 'look, this is mine, I leased it. This is now mine', like the **loggers who entered the fight forcefully*** [na pancada] . *We started to discover the value of staying put in our land, our lives, our habits. After the Reserve, now we're sought after by everyone and by authorities, **free from the command of outsiders who claimed to be the owner of land and lives**."*
Francisco Carneiro, "Azeite", Vila Limeira, quoted in Aleixo & ATAMP (2011), p7. Our translation and added emphasis.

On the River Trombetas meanwhile, *ribeirinhos* lament the greater rights enjoyed by those living in QT compared to the PAE they live in, but also note that before the 1980s the two populations were considered one (see Nepomuceno et al 2019:129). At Acari *ribeirinho* community elder residents, when asked to recall life prior to the 1980s, would normally say something along the lines of "everyone was the same", or, "before, that quilombola he was called a *ribeirinho*"; that there was "no distinction" between

communities now identifying as quilombola, and those nowidentifying as *ribeirinho*. As one said:

"They [quilombolas] didn't exist. But when they discovered the right to be quilombolas, now what did they do? … the quilombolas have their rights now. There used to be no [quilombola/ribeirinho] *distinction. Now that* [the rights] *arrived, they went and found them. There's lots of prejudice, isn't there? Then they went and they found out."* [quote taken from Nepomuceno et al 2019:129]

Nepomucento et al 2019 also reported that *ribeirinhos* emphasize shared practices, social conditions and land use, in the past and today, with quilombola communities: *"As to how they live and make their homes, how they eat together, it's the same thing [as us], there is almost no difference. In relation to the land, there is none. The same work that they do, we do as well. There is no difference"* one informant told us, after returning from living for nine years in a quilombola community.

References

Abel, M. (2022). The Struggle For Health: Medical Brokerage and the Power of Care in Brazil's Amazon Estuary. *Cultural Anthropology*, 37(3), 421-449.

Aleixo, J. and Associação dos Produtores Agroextrativistas da Assembleia de Deus do Rio Ituxi (APADRIT). (2011). *Memorial da luta pela Reserva Extrativista do Ituxi em Lábrea-AM: Registro da mobilização social, organização comunitária e conquista da cidadania na Amazônia.* Brasília: Instituto Internacional de Educação do Brasil (IIEB). https://iieb.org.br/wp-content/uploads/2019/02/public_ieb_Resex_medio_purus.pdf

Aleixo, J. and Associação dos Trabalhadores Agroextrativistas do Médio Purus (ATAMP). (2011). *Memorial da Luta pela Reserva Extrativista do Médio Purus em Lábrea-AM: Registro da mobilização social, organização comunitária e conquista de cidadania na Amazônia*. 94pp. Brasília: Instituto Internacional de Educação do Brasil (IEB).
https://iieb.org.br/wp-content/uploads/2019/02/public_ieb_Resex_medio_purus.pdf

Almeida, V. F. D., Schweickardt, J. C., Reis, A. E. S., & Vieira Moura, G. P. D. S. (2022). Caminhos da população ribeirinha no acesso à urgência e à emergência: desafios e potencialidades. *Interface-Comunicação, Saúde, Educação*, *26*, e210769.

Benzeev, R. (2022) Tropical forest restoration and land rights in Brazil. Ph.D. dissertation. University of Colorado Boulder.

Benzeev, R., Zhang, S., Rauber, M. A., Vance, E. A., & Newton, P. (2023). Formalizing tenure of Indigenous lands improved forest outcomes in the Atlantic Forest of Brazil. *PNAS nexus*, 2(1), pgac287.

Brown, W. (2006). *Regulating Aversion: Tolerance in the Age of Identity and Empire*. Princeton University Press.

Bustos, M. F., Hall, O., Niedomysl, T., & Ernstson, U. (2020). A pixel level evaluation of five multitemporal global gridded population datasets: a case study in Sweden, 1990–2015. *Population and Environment*, *42*, 255-277.

Butt, N., Lambrick, F., Menton, M., & Renwick, A. (2019). The supply chain of violence. *Nature Sustainability*, 2(8), 742-747.

Campos-Silva, J. V., & Peres, C. A. (2016). Community-based management induces rapid recovery of a high-value tropical freshwater fishery. *Scientific Reports*, 6(1), 34745.

Chacón-Montalván, E. A., Taylor, B. M., Cunha, M. G., Davies, G., Orellana, J. D., & Parry, L. (2021). Rainfall variability and adverse birth outcomes in Amazonia. *Nature Sustainability,* 4(7), 583-594.

Dagnino, R., Bueno, M., D'Antona, Á., & Pereira, H. (2013). População dentro de unidades de conservação federais no estado do Amazonas, Brasil. *Olam: Ciência & Tecnologia*, *13*(2).

de Castro, F. (2012). Multi-scale environmental citizenship. In: *Environment and citizenship in Latin America: natures, subjects and struggles*. Berghahn Books, Oxford,

UK, p. 39.

Dodd, L. M. (2020). Aspiring to a good life: rural–urban mobility and young people's desires in the Brazilian Amazon. *The Journal of Latin American and Caribbean Anthropology, 25*(2), 283-300.

Fries, B., Guerra, C. A., García, G. A., Wu, S. L., Smith, J. M., Oyono, J. N. M., ... &

Dolgert, A. J. (2021). Measuring the accuracy of gridded human population density surfaces: A case study in Bioko Island, Equatorial Guinea. *PLoS One*, *16*(9), e0248646.

Garnelo, L., Parente, R. C. P., Puchiarelli, M. L. R., Correia, P. C., Torres, M. V., &

Herkrath, F. J. (2020). Barriers to access and organization of primary health care services for rural riverside populations in the Amazon. *International Journal for Equity in Health*, *19*, 1-14.

Gonçalves, E. & Ferreira, P. (2020). Narcotráfico, garimpo, pesca e caça ilegal e contrabando de madeira se unem na Amazônia. Globo. https://oglobo.globo.com/brasil/noticia/2022/06/narcotrafico-garimpo-pesca-e-cacailegal-e-contrabando-de-madeira-se-unem-na-amazonia.ghtml

Hanberry, B. B. (2022). Imposing consistent global definitions of urban populations with gridded population density models: Irreconcilable differences at the national scale. *Landscape and Urban Planning*, *226*, 104493.

Hierink, F., Boo, G., Macharia, P. M., Ouma, P. O., Timoner, P., Levy, M., ... & Ray, N. (2022). Differences between gridded population data impact measures of geographic access to healthcare in sub-Saharan Africa. *Communications Medicine*, 2(1), 117.

IBGE (Instituto Brasileiro de Geografia e Estatística) (2010). Manual do Recenseador [CD-1.09] do Censo Demografico 2010.

https://biblioteca.ibge.gov.br/index.php/biblioteca-catalogo?id=52601&view=detalhes

IBGE (Instituto Brasileiro de Geografia e Estatística) (2016). Grade Estatistica. Available at: https://geoftp.ibge.gov.br/recortes_para_fins_estatisticos/grade_estatistica/censo_2010/grade_estatistica.pdf

Joppa, L. N., & Pfaff, A. (2009). High and far: biases in the location of protected areas. *PloS one*, 4(12), e8273.

el Kadri, M. R. E., Santos, B. S. D., Lima, R. T. D. S., Schweickardt, J. C., & Martins, F. M. (2019). Unidade básica de saúde fluvial: Um novo modelo da atenção básica para a Amazônia, Brasil. *Interface-Comunicação, Saúde, Educação*, *23*, e180613.

Kere, E. N., Choumert, J., Motel, P. C., Combes, J. L., Santoni, O., & Schwartz, S. (2017). Addressing contextual and location biases in the assessment of protected areas effectiveness on deforestation in the Brazilian Amazônia. *Ecological Economics*, 136, 148-158.

Landscan (2023) About Landscan webpage. Available at: https://landscan.ornl.gov/about

Lobo, M. S. D. C., & Cardoso, M. L. D. M. (2023). Lessons from urgent times: the experience of Yanomami health care then and now. *Cadernos de Saúde Pública*, 39, e00065623.

Luna, L. (2009). "Fazer viver e deixar morrer": a má-fé da saúde pública no Brasil." Chapter in: *SOUZA, Jessé. A ralé brasileira: quem é e como vive. Belo Horizonte: Editora da UFMG*.

Machado, C. (2020). Comunicação: Manuel Siqueira, presidente da ASPROC. Instituto Juruá. ttps://institutojurua.org.br/manuel-siqueira-presidente-da-asproc/

Mathews, M. C. (2021). How village leaders in rural Amazonia create bonding, bridging, and linking social capital configurations to achieve development goals, and why they are so difficult to maintain over time. *World Development*, *146*, 105541.

Mathews, M. C., & Schmink, M. (2015). "Differentiated citizenship" and the persistence of informal rural credit systems in Amazonia. *Geoforum*, 65, 266-277.

NASA (2018) Gridded population of the World (GWPv4). Documentation. Nasa Socieconomic Data and Applications Center. Available at: https://sedac.ciesin.columbia.edu/downloads/docs/gpw-v4/gpw-v4-documentationrev11.pdf

Nascimento, J. T. D. (2017). Ensino Médio presencial com mediação tecnológica numa escola ribeirinha do Amazonas. Masters thesis in Social Services, Federal University of Amazonas (UFAM). https://tede.ufam.edu.br/handle/tede/6115

Nepomuceno, Í., Affonso, H., Fraser, J.A. & Torres, M. (2019). Counter-conducts and the green grab: Forest peoples' resistance to industrial resource extraction in the Saracá-Taquera National Forest, Brazilian Amazonia. *Global Environmental Change*, 56, 124–133.

Newton, P., Kinzer, A. T., Miller, D. C., Oldekop, J. A., & Agrawal, A. (2020). The number and spatial distribution of forest-proximate people globally. *One Earth*, 3(3), 363-370.

Parry, L., Day, B., Amaral, S., & Peres, C. A. (2010). Drivers of rural exodus from Amazonian headwaters. *Population and Environment*, 32, 137-176.

Parry, L., Barlow, J., & Pereira, H. (2014). Wildlife harvest and consumption in Amazonia's urbanized wilderness. *Conservation Letters*, *7*, 565-574.

Parry, L., Davies, G., Almeida, O., Frausin, G., Moraés, A. de, Rivero, S., Filizola, N. & Torres, P. (2018). Social vulnerability to climatic shocks is shaped by urban

accessibility. *Ann. Am. Assoc. Geogr.*, 108, 125–143.

Parry, L., Radel, C., Adamo, S. B., Clark, N., Counterman, M., Flores-Yeffal, N., ... & Vargo, J. (2019). The (in) visible health risks of climate change. *Social Science & Medicine*, *241*, 112448.

Pereira, H. C., Nascimento, A. C. S. D., Moura, E. A. F., Corrêa, D. S. S., & Chagas, H. C. D. (2022). Migração rural-urbana por demanda educacional no Médio Solimões, Amazonas. *Revista Brasileira de Educação*, *27*, e270029.

Pereira, A. S. A., Dos Santos, V. J., do Carmo Alves, S., Amaral, A., Da Silva, C. G., & Calijuri, M. L. (2022). Contribution of rural settlements to the deforestation dynamics in the Legal Amazon. *Land Use Policy*, 115, 106039.

Perez, F. & Filho, H.B. (2024). Tiros, droga em submarino: PCC, milícia e piratas disputam rios na Amazônia. UOL. https://noticias.uol.com.br/cotidiano/ultimasnoticias/2024/01/03/piratas-faccoes-milicias-disputam-rios-amazonia.htm

Plano de Manejo RESEX Unini 2014. Instituto Chico Mendes de Conservação da Biodiversidade (ICMBio) https://www.gov.br/icmbio/pt-br/assuntos/biodiversidade/unidade-de-conservacao/unidades-de-biomas/amazonia/lista-de-ucs/resex-rio-unini

Plano de Gestão da RDS Amanã 2019. Secretaria de Estado do Meio Ambiente - Sema/AM http://meioambiente.am.gov.br/reserva-de-desenvolvimento-sustentavelamana/

Porro, R., & Porro, N. S. M. (2022). State-led social and environmental policy failure in a Brazilian forest frontier: Sustainable Development Project in Anapu, Pará. *Land Use Policy*, 114, 105935.

Santos, M. P., & Cunha, V. H. D. (2023). Dinâmicas da violência no estado do Amazonas (Publicação Preliminar). Instituto de Pesquisa Econômica Aplicada (IPEA). https://repositorio.ipea.gov.br/handle/11058/11983

Sorichetta, A., Hornby, G. M., Stevens, F. R., Gaughan, A. E., Linard, C., & Tatem, A. J. (2015). High-resolution gridded population datasets for Latin America and the Caribbean in 2010, 2015, and 2020. *Scientific Data*, 2(1), 1-12.

Staeheli, L.A. (2011). Political geography: Where's citizenship? *Progress in Human Geography*, 35, 393–400.

Stevens, F. R., Gaughan, A. E., Linard, C., & Tatem, A. J. (2015). Disaggregating census data for population mapping using random forests with remotely-sensed and ancillary data. *PloS one*, *10*(2), e0107042.

le Tourneau, F. M., & Greissing, A. (2010). A quest for sustainability: Brazil nut gatherers of São Francisco do Iratapuru and the Natura Corporation. *Geographical Journal,* 176(4), 334-349.

Viana, V., Adeodato, S., Correa, E., Villares, L., Costa, M., Solidade, V., Salviati, V., and Lira, S. (2021). Programa Bolsa Floresta: Trajetória, lições e desafios de uma política pública inovadora para a Amazônia (Fundação Amazônia Sustentável).

Wittman, H. (2010). Agrarian Reform and the Environment: Fostering Ecological Citizenship in Mato Grosso, Brazil. *Canadian Journal of Development Studies / Revue canadienne d'études du développemen*t, 29, 281–298.

WorldPop (2024) WorldPop methods webpage summarizing the approach to generate the population product. Available at: https://www.worldpop.org/methods/

Xu, Y., Ho, H. C., Knudby, A., & He, M. (2021). Comparative assessment of gridded population data sets for complex topography: a study of Southwest China. *Population and Environment*, *42*, 360-378.

Yanai, A.M., Graça, P.M.L. de A., Escada, M.I.S., Ziccardi, L.G. & Fearnside, P.M. (2020). Deforestation dynamics in Brazil's Amazonian settlements: Effects of landtenure

concentration. *Journal of Environmental Management*, 268, 110555.

Zanatto, V. G., & Rosa, P. C. Strategies for territorial regulation and use of common resources: challenges to shared management in the Amanã Sustainable Development Reserve–AM.

Appendix S10. Gridded population dataset products used to estimate resident population of forest citizens in qualifying territories

Data product	Developer	Details	Spatial Resolution	Adjusted to country totals?	Years available
Statistical grid (grade estatística)	IBGE	Based on Brazil's demographic census of 2010	Rural areas: 1 km; urban areas: 200 m	Yes	2010 (became available in 2016)
Landscan (global)	Department of Energy's Oak Ridge National Laboratory (ORNL).	None	1 km	No	2010, 2020
Gridded population	NASA	Version 4, adjusted to 2015 UN World Population Prospects country totals	1km	Yes	2010, 2022
World Pop	University of Southampton	Unconstrained corrected to 2015 UN World Population Prospects country totals	1km	Yes	2010, 2020

Note: documentation for products is available in the following websites (in the order of products in the table): https://geoftp.ibge.gov.br/recortes_para_fins_estatisticos/grade_estatistica/censo_2010/ ; https://landscan.ornl.gov/metadata ; https://www.earthdata.nasa.gov/data/catalog/sedac-ciesin-sedac-gpwv4-apct-wpp-2015-r11-4.11 ; https://hub.worldpop.org/geodata/listing?id=75

Appendix S11. Cross-validation of population estimates in the qualifying territories at the level of specific territories.

Name	Dagnino (2013)	Manag. plan	Population estimates in 2010				Validation against Dagnino (2013)				Validation vs Management Plan (median year = 2011)			
			IBGE grid (A)	Land Scan (B)	GPW (C)	WorldPop (D)	A	B	C	D	A	B	C	D
RESEX Medio Jurua	1899	1921	1733	485	1252	1787	-9%	-74%	-34%	-6%	-10%	-75%	-35%	-7%
RESEX Auati-Parana	1782	1376	1709	320	155	1013	-4%	-82%	-91%	-43%	24%	-77%	-89%	-26%
RESEX Baixo Jurua	682	643	247	374	338	427	-64%	-45%	-50%	-37%	-62%	-42%	-47%	-34%
RESEX Arapixi	291	610	618	902	213	725	112%	210%	-27%	149%	1%	48%	-65%	19%
RESEX Rio Jutai	1537	1221	1122	396	60	715	-27%	-74%	-96%	-53%	-8%	-68%	-95%	-41%
RESEX Rio Unini	1219	621	364	627	66	656	-70%	-49%	-95%	-46%	-41%	1%	-89%	6%
RESEX Lago do Capanã Grande	1089	1032	783	175	267	884	-28%	-84%	-76%	-19%	-24%	-83%	-74%	-14%
RESEX do Rio Iriri	NA	285	269	1327	21	225	NA	NA	NA	NA	-6%	365%	-93%	-21%
RESEX Catuá Ipixuna	NA	1457	1064	357	327	1263	NA	NA	NA	NA	-27%	-75%	-78%	-13%
RESEX Canutama	NA	780	506	761	84	630	NA	NA	NA	NA	-35%	-2%	-89%	-19%
RESEX Guariba	NA	0	0	10	2	16	NA	NA	NA	NA	0	9.95	1.68	16.19
RESEX Rio Gregório	NA	1100	966	424	307	1170	NA	NA	NA	NA	-12%	-61%	-72%	6%
RDS Amanã	NA	5448	3533	3735	794	4613	NA	NA	NA	NA	-35%	-31%	-85%	-15%
RDS Aripuanã	NA	0	79	34	1	117	NA	NA	NA	NA	79.00	34.33	0.59	117.17
RDS Bararati	NA	0	2	71	0	2	NA	NA	NA	NA	1.94	71.11	0.16	1.71
RDS Cujubim	NA	147	126	3463	22	213	NA	NA	NA	NA	-14%	2256%	-85%	45%

The validation columns (eight last columns) contain percent differences except for units #11, 14, 15 and 19, whose validation value is zero, so that absolute differences were computed.

Appendix S12. Continuation of cross-validation of population estimates in the target territories at the level of specific units

Name	Dagnino (2013)	Management Plan Pop.	Population estimates 2010				Validation vs Dagnino (2013)				Validation vs Management Plan			
			IBGE Grid (A)	LandScan ((B)	GPW (C)	WorldPop (D)	A	B	C	D	A	B	C	D
RDS Uacari	NA	1304	1476	743	286	1253	NA	NA	NA	NA	13%	-43%	-78%	-4%
RDS Juma	NA	1188	1123	702	184	1254	NA	NA	NA	NA	-6%	-41%	-84%	6%
RDS Matupiri	NA	0	0	255	10	27	NA	NA	NA	NA	-	255.24	9.98	26.53
RDS Rio Amapá	NA	1150	0	168	12	107	NA	NA	NA	NA	-100%	-85%	-99%	-91%
RDS Rio Negro	NA	2364	1098	4698	212	713	NA	NA	NA	NA	-54%	99%	-91%	-70%
RDS Uatumã	NA	1312	2316	1578	198	2215	NA	NA	NA	NA	77%	20%	-85%	69%
RDS Piagaçu – Purus	NA	4000	2664	5834	754	3684	NA	NA	NA	NA	-33%	46%	-81%	-8%
RDS Puranga Conquista	NA	1588	362	920	71	498	NA	NA	NA	NA	-77%	-42%	-96%	-69%
RDS Rio Madeira	NA	2927	1955	744	694	2245	NA	NA	NA	NA	-33%	-75%	-76%	-23%
RDSMamiraua	NA	5780	4315	9021	1448	6221	NA	NA	NA	NA	-25%	56%	-75%	8%
	**Average percent error**						**-13%**	**-28%**	**-67%**	**-8%**	**-22%**	**97%**	**-80%**	**-15%**

Note: the management plan of unit 21 originally referred to the family count. It was converted to individuals by assuming 3.8 individuals/family.

Appendix S13. Cross-validation of Management Plan population estimates between two years in three Sustainable Development Reserves (RDS) in Amazonas state

Name	Pop. in management plan, 2010 or circa	Pop. in management plan, 2020 or circa	Growth rate	Population estimates, 2010			Population estimates, 2020			Growth rate			Validation against 2020 Management Plan (or circa)			Growth rate validation		
				LandScan (B)	GPW (c)	WorldPop (D)	LandScan (B)	GPW (c)	WorldPop (D)	LandScan (B)	GPW (c)	WorldPop (D)	LandScan (B)	GPW (c)	WorldPop (D)	LandScan (B)	GPW (c)	WorldPop (D)
RDS Rio Amapá	1150	1550	35%	168	12	107	36	14	117	-79%	18%	10%	-98%	-99%	-92%	-327%	-48%	-72%
RDS Uatumã	1312	1644	25%	1578	198	2215	2,333	294	3,018	48%	48%	36%	42%	-82%	84%	89%	89%	43%
RDS Mamiraua	5780	6224	8%	9021	1448	6221	2,709	1,242	5,122	-70%	-14%	-18%	-56%	-80%	-18%	-1011%	-286%	-330%
**Average percent error**													**-37%**	**-87%**	**-9%**	**-416%**	**-82%**	**-119%**
**Average percent error, without RDS Mamiraua**													**-28%**	**-91%**	**-4%**	**-119%**	**21%**	**-14%**

Note: the years for which data were available in the management plan were: 2010 and 2018 for RDS Rio Amapá, 2007 and 2017 for RDS Uatumã and 2011 and 2019 for Mamiraua.

Appendix S14. Standard deviations of population growth rates (2010 to 2020) by territory and gridded population product

Territorial category / Data product	LandScan	GPW	WorldPop
Indigenous Lands	2400.88	0.38	0.30
Quilombola Territories	98.13	1.55	0.44
Ecological Settlement Projects	3320.97	10.77	0.38
Sustainable use (RESEX/RDS)	89.70	0.44	0.91

Note: standard deviations calculated at the level of particular territorial areas (polygons)

Appendix S15. Population growth (number of permanent residents) estimates at the level of territories

Territory / product		IBGE grid (A)	LandScan (B)	GPW v4 (C)	WorldPop (D)	Censuses (E)
Indigenous Lands	**2010**	286,389	261,964	232,794	325,000	323,947
	**2020**	NA	294,885	285,097	404,950	403,209
	Growth	**NA**	**13%**	**22%**	**25%**	**24%**
Quilombola Territories	**2010**	52,583	49,124	42,714	55,421	NA
	**2020**	NA	39,354	48,182	61,387	80,860
	Growth	**NA**	**-20%**	**13%**	**11%**	**NA**
RESEX and RDS sustainable use reserves	**2010**	124,759	126,982	102,127	168,929	NA
	**2020**	NA	133,973	113,291	193,608	NA
	Growth	**NA**	**6%**	**11%**	**15%**	**NA**
Ecological Settlement Projects	**2010**	327,935	145,594	236,816	329,951	NA
	**2020**	NA	289,953	282,265	394,157	NA
	Growth	**NA**	**99%**	**19%**	**19%**	**NA**
Total forest citizen population	**2010**	791,666	583,664	614,450	879,301	323,947
	**2020**	NA	758,165	728,835	1,054,102	484,069
	Growth	**NA**	**29.9%**	**18.6%**	**19.9%**	**49.4%**
